# Spread of hospital-acquired infections: A comparison of healthcare networks

**DOI:** 10.1371/journal.pcbi.1005666

**Published:** 2017-08-24

**Authors:** Narimane Nekkab, Pascal Astagneau, Laura Temime, Pascal Crépey

**Affiliations:** 1 Laboratoire MESuRS, Conservatoire National des Arts et Métiers, 292 Rue Saint-Martin, Paris, France; 2 Institut Pasteur, Cnam, Unité PACRI, 25–28, rue du Docteur Roux, Paris, France; 3 Ecole des Hautes Etudes en Santé Publique, Département d'Epidémiologie et de Biostatistiques, 15 Avenue du Professeur-Léon-Bernard, Rennes, France; 4 Centre de prévention des infections associées aux soins (C-CLIN), APHP, Paris, France; 5 Faculté de médecine Pierre et Marie Curie, Sorbonne Universités, Paris, France; 6 UMR190, Emergence des Pathologies Virales, Marseille, France; 7 UPRES EA 7449 Reperes, Rennes, France; University of New South Wales, AUSTRALIA

## Abstract

Hospital-acquired infections (HAIs), including emerging multi-drug resistant organisms, threaten healthcare systems worldwide. Efficient containment measures of HAIs must mobilize the entire healthcare network. Thus, to best understand how to reduce the potential scale of HAI epidemic spread, we explore patient transfer patterns in the French healthcare system. Using an exhaustive database of all hospital discharge summaries in France in 2014, we construct and analyze three patient networks based on the following: transfers of patients with HAI (HAI-specific network); patients with suspected HAI (suspected-HAI network); and all patients (general network). All three networks have heterogeneous patient flow and demonstrate small-world and scale-free characteristics. Patient populations that comprise these networks are also heterogeneous in their movement patterns. Ranking of hospitals by centrality measures and comparing community clustering using community detection algorithms shows that despite the differences in patient population, the HAI-specific and suspected-HAI networks rely on the same underlying structure as that of the general network. As a result, the general network may be more reliable in studying potential spread of HAIs. Finally, we identify transfer patterns at both the French regional and departmental (county) levels that are important in the identification of key hospital centers, patient flow trajectories, and regional clusters that may serve as a basis for novel wide-scale infection control strategies.

## Author summary

Hospital-acquired infections (HAIs), including emerging multi-drug resistant organisms, threaten healthcare systems worldwide. Efficient containment measures of HAIs must mobilize the entire healthcare network. Thus, to best understand how to reduce the scale of potential HAI epidemic spread, we explore patient transfer patterns in the French healthcare system. We construct and compare the characteristics of three different patient transfer networks based on data on transfers of patients with diagnosed HAIs, suspected HAIs, or of all patients. Our analyses show that these healthcare networks, the patient populations that comprise them and the patient movement patterns are heterogeneous and centralized. Despite the differences in patient populations, the HAI-specific and suspected-HAI healthcare networks have the same underlying structure as that of the general healthcare network. We identify key hospital centers, patient flow trajectories, at both the regional and department (county) level that may serve as a basis for proposing novel wide-scale infection control strategies.

## Introduction

The emergence and spread of multi-drug resistant organisms threatens healthcare systems worldwide.[1] This is particularly true concerning methicillin-resistant *Staphylococcus aureus*, vancomycin-resistant enterococci, and multi-resistant gram-negative bacteria such as carbapenemase-producing Enterobacteriaceae (CPE). Spread of CPE is now a global public health problem associated with patient transfers between healthcare facilities within the same country as well as across national borders, as shown in many recent studies.[2–7]

In recent years, patient transfer or referral data has been used to construct “healthcare networks” to propose innovative approaches for hospital infection prevention and control. Healthcare networks are cooperative healthcare systems where hospitals and other healthcare centers are linked by shared patients through secondary transfers or referral.[8, 9] Rather than being exclusive to one sole hospital, as Ciccolini et al. argue, the extent of hospital-acquired infection (HAI) spread is dependent on the healthcare network connected by inter-institutional patient transfers.[8] Heterogeneous hospital patient populations and the interactions that occur between them and with the community are important in the understanding of the spatial spread of HAI between hospitals across geographic regions.[9]

As early as 2007, studies applied more complex social network analysis approaches to reconstructed healthcare networks in order to demonstrate that infection control measures that take into account network properties can decrease the risk for outbreaks.[8, 10] Lee et al. consider network properties to assess the individual influence of different hospitals and the impact of hospital proximities on HAI spread on a regional scale.[11] Many studies show that healthcare networks display a community structure.[8, 12–14] Network analysis is especially effective in the identification of sensor hospitals for surveillance of HAIs.[15, 16] In addition, mathematical models of healthcare networks may serve to inform decision-makers on enhanced coordinated regional and national approaches to infection control strategies, in a context where increasingly centralized healthcare systems favor the spread of HAIs.[8, 15, 17]

Although national healthcare networks are informative regarding novel HAI control strategies, the impact of reconstructing these networks based on a general patient population rather than a HAI-diagnosed patient population has rarely been addressed. In this study, we assess and compare French healthcare networks based on either patients diagnosed with HAIs or the general patient population, in order to better understand the potential implications in terms of HAI spread predictions. To that aim, we perform social network analyses to describe the different patient flow patterns, network topology characteristics, and community clustering structure.

## Results

We analyzed and compared three different networks built using transfer data from an exhaustive database of all hospital discharge summaries in France in 2014: (1) a network based on transfers of patients with diagnosed HAI (HAI-specific network); (2) a network based on transfers of patients with suspected HAI (suspected-HAI network); and (3) the network of all patient transfers (general network).

### Characteristics of healthcare networks

More than 10 million hospital transfers were recorded in France in 2014, for a total of 2.3 million transferred patients, creating a hospital network of 2063 hospitals (nodes) and 50026 patient trajectories (edges) linking them (Table 1). Patients with a HAI-specific diagnosis created a healthcare network of 1266 hospitals and 3722 connections for 13627 patient transfers. A larger population of patients suspected to have an HAI infection formed a healthcare network of 1975 hospitals and 18812 connections for a total of 128681 patient transfers. With the increasing number of patient transfers, the networks increased from an average 5.88, 19.05, and 48.05 average connections per hospital (average degree $\bar{k}$) and an average 2.31, 4.92 to 14.02 transfers per connection (average strength $\bar{s}$) for the HAI-specific, suspected-HAI, and general healthcare networks respectively (Table 1).

**Table 1:** Networks characteristics of the French healthcare networks.

Network Characteristics	General Network	Suspected-HAI Network	HAI-Specific Network
Patients	2300728	394859	21279
Patient Transfers	1033239	128681	13627
Hospitals	2063	1975	1266
Patient Trajectories*	50026	18812	3722
Average Edge Weight**	14.02	4.92	2.31
Average Degree***	48.50	19.05	5.88
Average In-Degree	24.25	9.53	2.94
Average Out-Degree	24.25	9.53	2.94
Average Betweenness***	5292.32	6338.81	3824.91
Average Edge Betweenness	301.27	852.23	1556.94
Average Closeness***	0.00016	0.000074	0.000032
Diameter	30	64	47
Average Path Length	2.99	3.63	5.23
Global Clustering Coefficient	0.23	0.16	0.08
Density	0.012	0.005	0.002

* Also referred to as edges, they represent the sum number of connections between the hospitals

** The average number of patients per trajectory

*** Measures of node (or hospital) centrality

Overall, the three networks displayed “scale-free” and “small-world” characteristics that indicated the presence of a small number of very highly connected hospitals with high degrees, referred to as “hubs.” Analyses of the degree, strength, and shortest path length distributions in addition to the small-world characteristics of the healthcare networks are discussed in S1–S3 Texts and S1–S7 Figs. Compared to random networks, we also showed the general network was more clustered and efficient in transferring patients (S4 Text, S1 Table). We identified several high degree hospitals in all three networks with a consistent outlier–the Assistance Publique—Hôpitaux de Paris (AP-HP)–a conglomerate of 39 hospitals predominately in Paris and the Ile-de-France region represented as one hospital code in our database.[18] AP-HP also acted as the most important intermediary hospital system in the networks due to having the highest betweenness centrality measure.

The hospitals involved in the patient transfers recorded in the three networks were of various types, including private rehabilitation and postoperative care facilities, acute-care hospitals or clinics, and hospital centers (Table 2). However, the majority of hubs, defined as the top 5% of hospitals by their degree, were large hospitals providing both acute and postoperative or rehabilitation care (67%, 65%, and 88% in the general, suspected-HAI, and HAI-specific networks respectively). In addition, in the general and suspected-HAI networks, hubs were mostly acute-care hospitals or clinics, hospital centers, or university hospitals centers, with many concentrated in the Ile-de-France, Marseille, and Lyon metropoles (31%, 33%, 28%, and 32%, 28%, 30% respectively). In contrast, university hospital centers rather than acute-care facilities dominated the hub hospitals of the HAI-specific network, representing 48% of hubs (Table 2). The hub university healthcare centers, which provided highly specialized services, included the AP-HP, Hospices Civils de Lyon, and the Assistance Publique—Hôpitaux de Marseille (AP-HM); among them there were also university hospitals of other major cities in France.

**Table 2:** Healthcare facility types among the general, suspected-HAI, and HAI-specific networks and their hub hospitals.

Health Facility Type		General		Suspected-HAI		HAI-Specific	
		All facilities	Among hubs* (N = 103)	All facilities	Among hubs* (N = 99)	All facilities	Among hubs* (N = 63)
Type 1**	SSR***	38.00%	0.97%	37.22%	2.02%	35.31%	1.69%
	MCO**** & SSR	36.74%	66.99%	38.13%	64.65%	44.63%	88.14%
	MCO	25.25%	32.04%	24.66%	33.33%	20.06%	10.17%
Type 2^†^	Private hospitals authorized to provide SSR services	30.63%	0	29.90%	1.03%	30.76%	1.72%
	Acute-care hospitals or clinics	28.72%	30.69%	28.83%	31.96%	25.04%	5.17%
	Hospital centers	23.50%	32.67%	24.44%	27.84%	30.29%	31.03%
	Local hospitals	10.75%	0	10.77%	0	6.76%	0
	University hospital centers^††^	1.47%	27.72%	1.53%	29.90%	2.38%	48.28%
	Nursing home	1.27%	0.99%	1.22%	0	1.35%	0
	Cancer centers	0.93%	3.96%	0.97%	4.12%	1.27%	3.45%
	Other health facilities practicing under the healthcare law	0.59%	0	0.56%	2.06%	0.48%	1.72%
	Armed forces hospitals	0.44%	2.97%	0.46%	3.09%	0.72%	8.62%
	Long-term care hospitals	0.39%	0.99%	0.41%	0	0.24%	0
	Other facilities for mental health	0.39%	0	0.26%	0	0.08%	0
	Medical homes for handicapped adults	0.34%	0	0.26%	0	0.32%	0
	Hospital centers specialized in mental health	0.24%	0	0.10%	0	0.08%	0
	Home care facilities	0.20%	0	0.15%	0	0.16%	0
	Outpatient dialysis centers	0.10%	0	0.10%	0	0	0
	Home care or outpatient care for the handicapped	0.05%	0	0.05%	0	0.08%	0

The percent of different health facilities in the networks by Type 1 and Type 2 classification are given.

* Hubs are defined as facilities that comprise the top 5% of facilities by degree

** Type 1 refers to categorization of the general activities performed in the facility

*** SSR = postoperative and rehabilitation care (soins de suite et de réadaptation)

**** MCO = medical, surgery, and/or obstetrics care (médecine—chirurgie—obstétrique)

^†^ Type 2 refers to the categorization of the facilities by hospital type or specialized services

^††^ Often referred to as regional hospital centers (centre hospitalier régionale)

**Fig 1:**
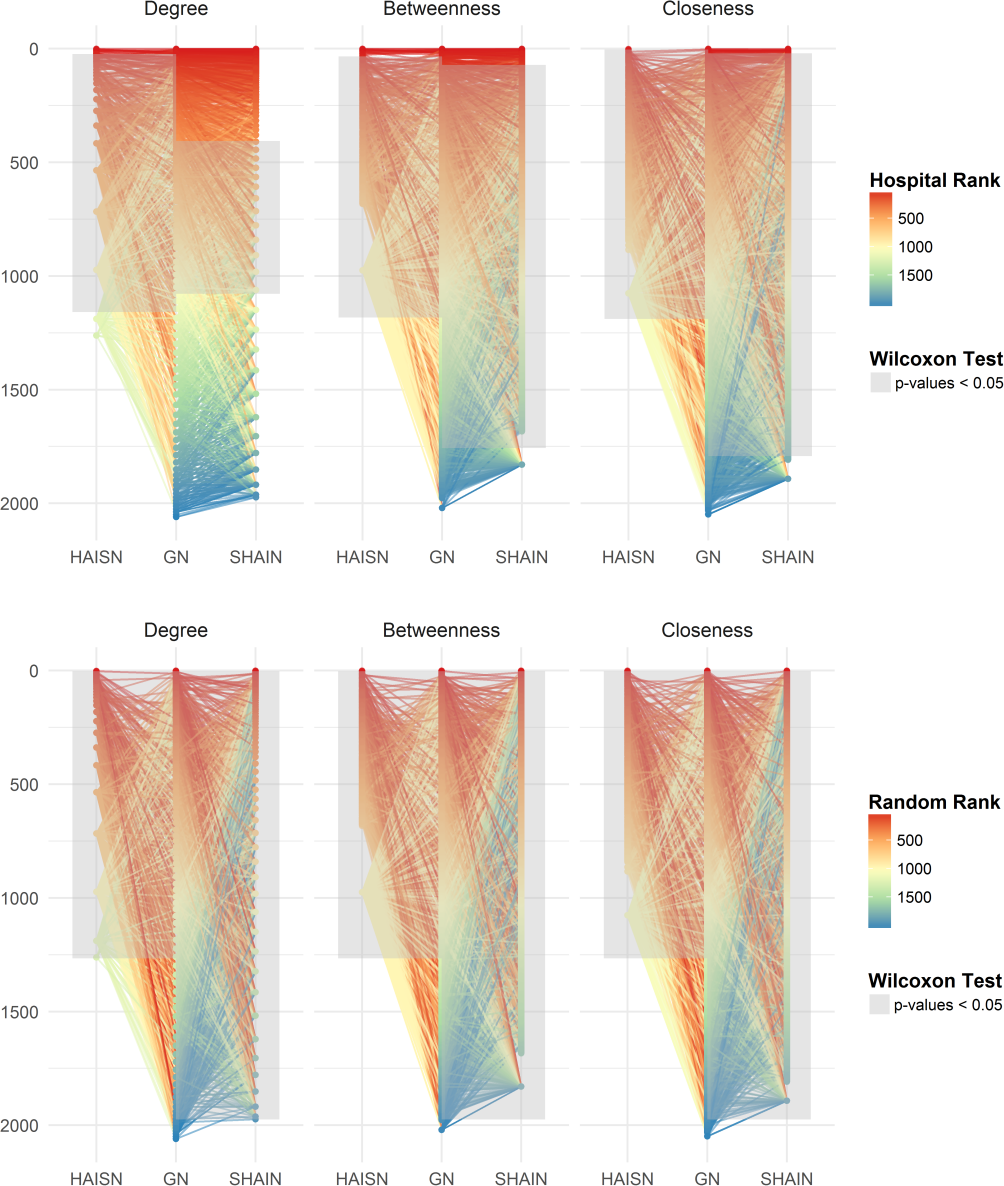
Hospital rankings by degree, betweenness, and closeness across the networks. Hospitals in the HAI-specific network (HAISN) (n = 1266), suspected-HAI network (SHAIN) (n = 1975), and general network (GN) (n = 2063) are displayed vertically and plotted against their ranking by degree, betweenness, and closeness centrality measures (top row). Only the hospitals shared between the HAISN and GN or the SHAIN and GN were linked. The color gradient refers to the hospital ranking for each centrality measure and the line colors correspond to the rankings of the hospitals in the GN. We tested the differences in rankings by Wilcoxon rank sum test of an increasing subset of hospital degrees starting from the highest rank and adding each consecutive rank and retesting. The grey area represents the range where the HAISN or SHAIN differed from the general network hospital rankings. We chose rankings at random for the hospital degrees, betweenness, and closeness centrality measures for comparison (bottom row). All random rankings were statistically different across the centrality measures between the HAISN and GN and the SHAIN and GN shared hospitals.

To better understand the role of hub hospitals across the networks, the shared hospitals between the networks were ranked based on their degree, closeness, and betweenness (Fig 1). Overall, when comparing the degree, betweenness, and closeness, the hospital rankings did not differ between the complete set of 1266 HAI-specific network hospitals and these same hospitals in the general network (p = 0.81, p = 1, p = 0.99 respectively, Wilcoxon rank sum test), or between the 1975 suspected-HAI network hospitals and the same hospitals in the general network (p = 0.99, p = 1, p = 0.99, Wilcoxon rank sum test). For comparison and illustration purposes, we showed that random rankings for degree, betweenness, and closeness of all hospitals differed significantly between patient specific networks and the general network (p < 0.05 respectively, Wilcoxon rank sum test) (Fig 1).

Suspecting that the differences between rankings might exist between subsets of hospitals, we tested the differences between rankings on an increasing subset of shared hospitals, starting with the highest rank, adding the next ranked hospital, and testing for significant differences. As a result, we determined the range of hospital rankings across the networks where the rankings significantly differed. We defined significant differences as Wilcoxon rank sum test p-values under the 5% alpha risk which we represent as a grey area in Fig 1. Distributions of these p-values are provided in S8 and S9 Figs. For the HAI-specific network, the range of statistically significant degree ranking differences were observed between the 24^th^ ranked hospital to the 1159^th^ ranking hospital. For the suspected-HAI network, statistically significant degree ranking differences were observed between the 405^th^ ranked hospital to the 1078^th^ ranked hospital.

For hospital rankings based on betweenness and closeness centrality measures, the hospitals ranked with highest and lowest centralities in the general network were also the hospitals ranked with highest and lowest centralities ranking in the HAI-specific and suspected-HAI networks. Even though hospital rankings of all hospitals did not differ, the majority did differ for betweenness ranks between the 33^rd^ highest ranking to the 1183^rd^ ranking in the HAI-specific network and the 71^st^ highest ranking to the 1757^th^ ranking in the suspected-HAI network (p < 0.05, Wilcoxon rank sum test). Closeness rankings differences were observed for almost all rankings after the first 3 rankings in the HAI-specific network and after the first 6 in the suspected-HAI network. The lack of statistically significant differences for the highest rankings may have been only due to insufficient power and for lowest hospital rankings due to a series of repeating small closeness values. With this method, we highlight that differences do exist for subsets of hospitals, but we also observe that the most highly connected hub hospitals were consistently highly connected across the networks, irrespective of the different patient population that connected them.

### What community structures in patient sharing are observed?

To further assess patient movement patterns in the networks, we investigated how our healthcare networks displayed “community” or hospital clustering structure. We compared hospital communities detected with two different community clustering algorithms: 1) the Greedy algorithm [19] that selected members of the communities to maximize the density of links between vertices as it reconstructed the network one vertex at a time and 2) the Map Equation algorithm [20], based on network structure-induced movement using a flow-based and information-theoretic method, detecting communities by measuring probability flows by taking into consideration the directionality and weight of the edges. In general, we detected fewer communities with the Greedy algorithm given that it seeks to maximize modularity–a value that measures the density of links inside communities by comparing the fraction of edges within the communities to the fraction in a random network; a maximum value of 1 corresponds to a network structure with the highest strength possible–as a result, the algorithm searched for the repartitions that maximized the density of the edges.[21–23] The Greedy algorithm considered pairwise interactions and the formation of the network whereas the Map Equation considered the interdependence of links and the dynamics of an already formed network.

For each network, we calculated the modularity, the number of communities, community size, and average community clustering distance using the Greedy and Map Equation community detection algorithms (Table 3). For each community, the pairwise clustering distance was calculated as the average geographic distance between all pairs of hospitals of the same community in kilometers. Compared to the general healthcare network, the patient-specific networks had more communities. In the HAI-specific network, there were on average 35.17 hospitals per community (SD = 44.31) and 31.40 kilometers between pairs of hospitals in the same community (SD = 25.60 km). In the larger networks, the larger community sizes resulted in a higher average distance between community hospitals (41.60 km (SD = 34.71) and 39.01 km (SD = 45.63) for the suspected-HAI and general healthcare network respectively). For the Map Equation-based communities, as the number of communities decreased from the HAI-specific to suspected-HAI to the general healthcare network, the average community size and average community distance between hospitals of the same community increased (Table 3). Overall, the suspected-HAI network was more similar to the general network than the HAI-specific network in terms of community structure (S5 Text).

**Table 3:** Community clustering distance.

	General Network	Suspected-HAI Network	HAI-Specific Network
Map Equation algorithm			
Modularity	0.764	0.716	0.698
Number of communities	132	160	193
Average community size	15.63	12.34	6.56
Average community clustering distance (km)	30.51	23.63	22.86
Greedy algorithm			
Modularity	0.863	0.847	0.830
Number of communities	18	21	36
Average community size	114.61	94.05	35.17
Average community clustering distance (km)	39.01	41.60	31.40

Two community detection algorithms were used to assess community clustering for each network, both of which take into account weighted graphs. The Greedy algorithm, developed by Clauset et al.[19] optimized modularity; however, it applied only to non-directed graphs. The Map Equation[20] algorithm applied to directed graphs and detects communities based network structure-induced movement using a flow-based and information-theoretic method. Average community size refers to the average number of hospitals within a detected community. For each community, the clustering distance in kilometers was calculated as the average geographic distance between pairs of hospitals of the same community.

**Fig 2:**
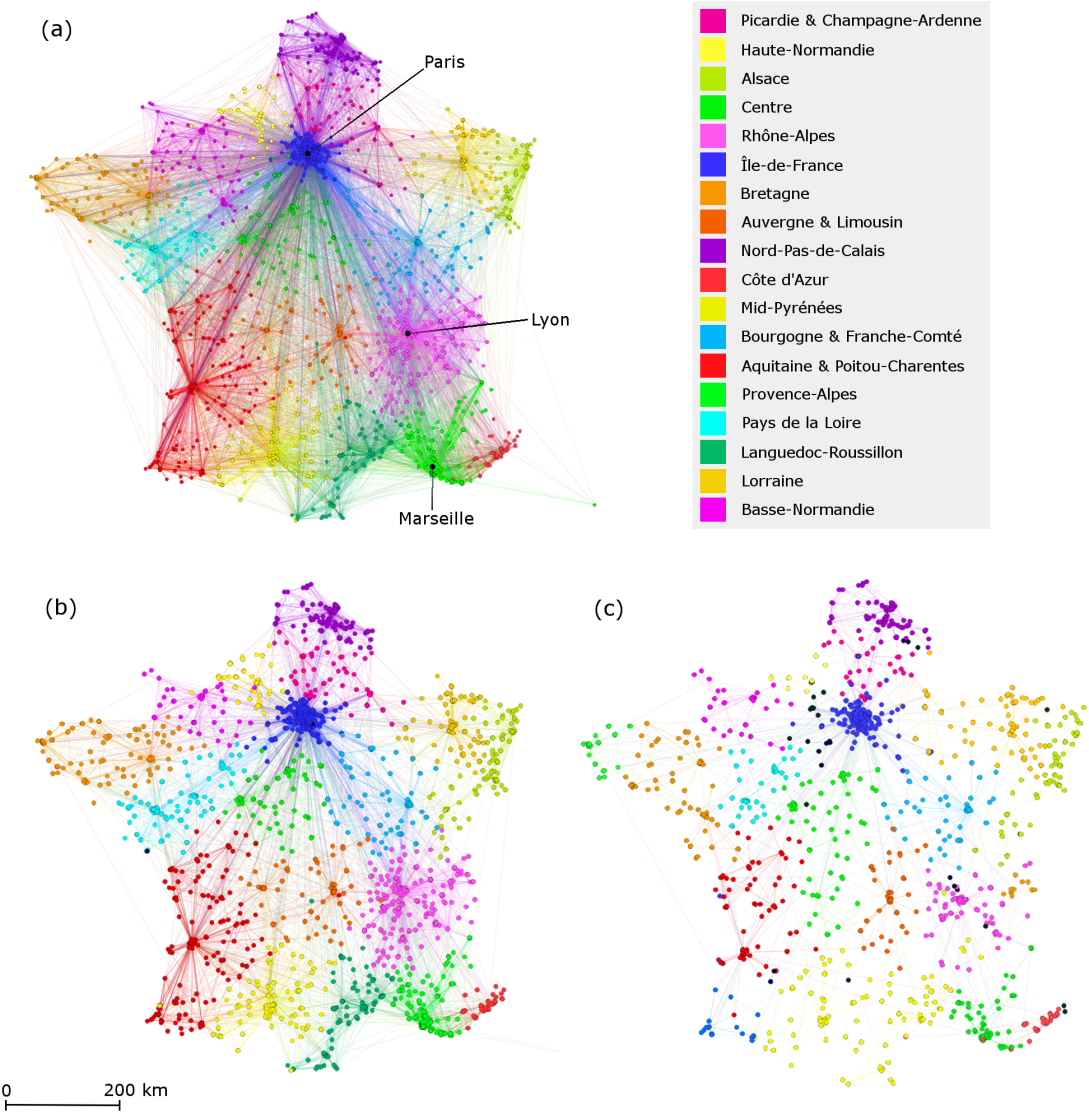
Regional clustering of communities detected with greedy algorithm. Network hospitals and patient trajectories of the healthcare network in France of (a) the general healthcare network, (b) the suspected-HAI healthcare network, and (c) the HAI-specific healthcare network. In the general healthcare network, 18 communities were detected by the community clustering algorithm. Four of the 18 communities identified by the algorithm combine hospitals from two regions each, such that the 22 geographical regions are mapped into 18 communities. The original 22 French metropolitan regions before they were reformed to 13 regions implemented in 2016 are shown to correspond to the 2014 data. For the HAI-specific and suspected-HAI networks, the algorithm detected a higher number of communities (36 and 21 communities respectively). The communities, which overlapped the same regional communities in the general network, were given the same color and the newly detected communities were given different colors.

The regional community clustering using the Greedy algorithm in the three networks are represented in Fig 2. The hospitals in communities were geo-localized, color-coded, and labelled across the networks according to the administrative region(s) they encompassed. We observed that the Greedy-based communities accurately reflected the French regional administrative structure (Fig 2). The identified community clusters formed hospitals communities in which most of the patients were shared between hospitals of the same region frequently centralized towards the hub acute-care centers, university hospital centers, and general hospital centers. On the other hand, the Map Equation-based communities displayed geographic community clustering at the French “departmental” or county level–the administrative division between the administrative region and the municipalities, similar to “counties” or “districts”; of which 96 departmental divisions are present in continental France. The vast majority of these departmental-level community clusters were acute-care centers followed by university hospitals centers and long-term care facilities.

### What are the patterns of patient transfer between communities?

**Fig 3:**
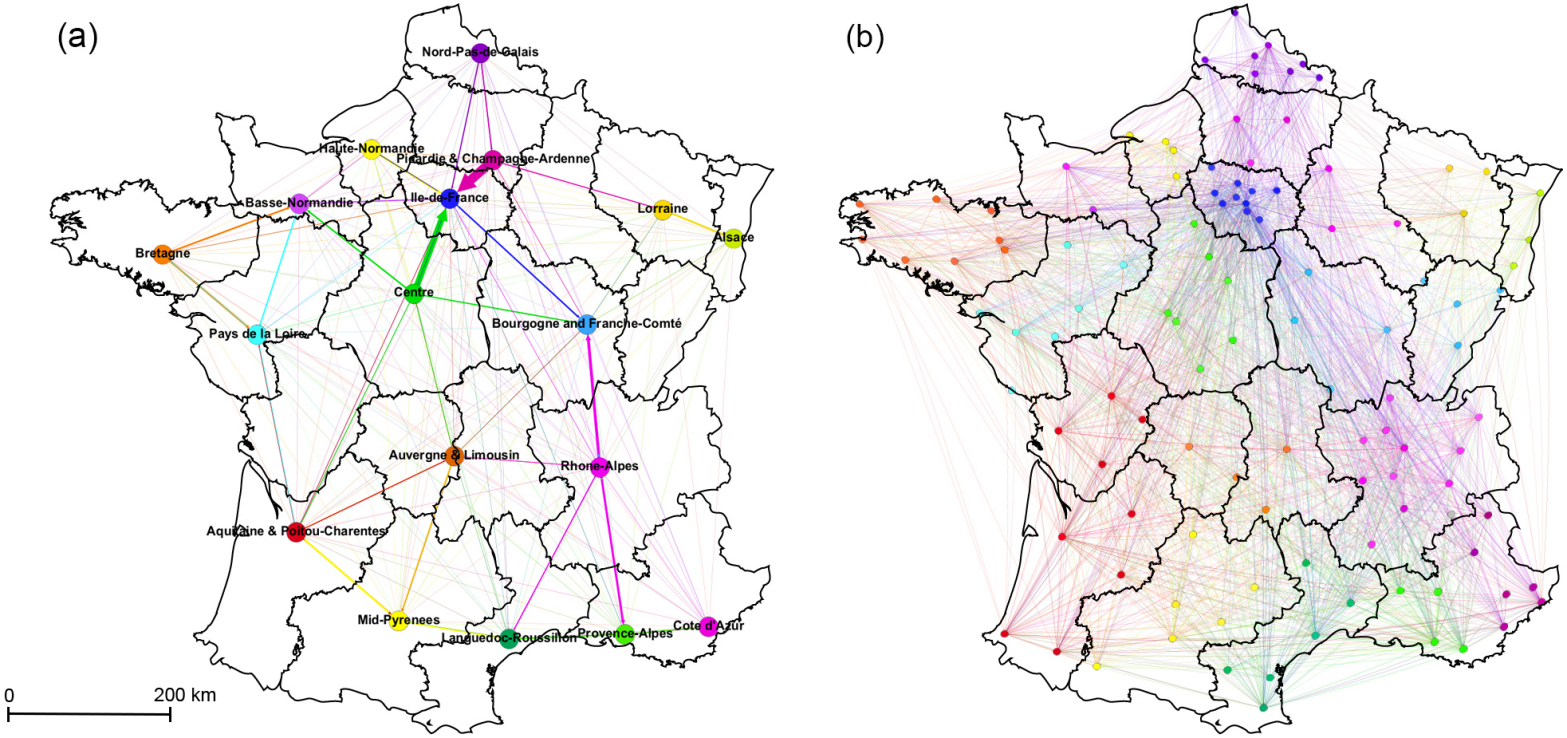
The intercommunity networks of patient transfers. (a) The intercommunity network from the 18 detected general patient network Greedy-based communities named based on the French metropolitan regions they encompass. Edge size and color indicate the source community and number of patients discharged. (b) The intercommunity network from 113 Map Equation communities detected in the general network. The nodes of the networks represent the geographical center of hospitals within the shared community.

To further understand the community structure, we constructed intercommunity networks by combining patient flows between hospitals of the same community and across communities. The Greedy-based intercommunity network was composed of 18 nodes representing the sum of all patient transfers that occurred between hospitals of each community with 306 regional transfer trajectories (Fig 3A). Out of the 22 French metropolitan regions in 2014, 4 pairs of 8 metropolitan regions were combined in this intercommunity network (Picardie and Champagne-Ardenne, Auvergne and Limousin, Aquitaine and Poitou-Charentes, and Bourgogne and Franche-Comté). The network was completely connected. All regional communities were connected to one another with an average of 4590 patients moving within these intercommunity trajectories over the year. Certain trajectories played a larger role in patient movement, notably Ile-de-France which admitted the largest number of patients from neighboring regions Picardie and Champagne-Ardenne (4772 transfers) and Centre (3205 transfers) where healthcare hubs were most concentrated. The subsequent largest traffic came from the Rhone-Alpes, the second largest regional center around the city of Lyon, which discharged patients to its neighboring regions (1482 transfers to neighboring Bourgogne and Franche-Comté and 1342 transfers to neighboring Provence-Alps respectively). Nonetheless, the greatest amount of transfers (93%) occurred within the communities themselves on average with up to 98% of transfers occurring within Ile-De France for instance. Although most of these transfers occurred within the communities, the regions remained highly interconnected and certain trajectories played an important role in the interregional and nation-wide movement of patients in France.

Building the intercommunity network where community affiliation was determined by the Map Equation algorithm allowed us to consider communities based on the directionality of patient flow, which was overlooked by the Greedy algorithm. The intercommunity network was composed of 113 community nodes with 3215 trajectories with an average degree of 57 and an average of 2597 patients moving between these connections (Fig 3B). Map Equation-based intercommunity communities demonstrated more comprehensive department-level patient flow. Communities were composed of hospitals from many different departments within and across regions; however, the majority of communities were of hospitals within the same department where most of the patient exchange occurred. Concerning the most important transfer routes with the highest traffic, discharged patients coming from many neighboring departments were preferentially going to hospitals in one or a few number of departments, indicating that there was interdepartmental centralization of patient movement. For example, a community composed of 200 hospitals from 9 Ile-de-France departments sent the largest number of transfers (3137 patients) to 28 hospitals of which 24 were from one department (Val-d’Oise). In exchange, this 28-hospital community sent back 2772 patients to the larger community. Overall, patient transfers in Map-Equation communities displayed departmental clustering, but also demonstrated asymmetric movements of patients, concentrating towards small communities of hospitals usually in one department, illustrating the different nature of the communities.

Patient sharing patterns and community clustering in the networks were also analyzed based on patient age groups in which new communities were identified (S6 Text, S10–S12 Figs). Moreover, analysis of monthly temporal dynamics of the networks showed that monthly communities may be less clustered and patients may not visit all of the hospitals each month but they still retained the same regional patient sharing patterns seen in the annual network (S7 Text).

### Do HAI-diagnosed patients have specific transfer flows in the healthcare network?

Having assessed the role of hospitals, hospital communities, and patient trajectories in each network, we considered if the differences in the patient-specific networks and the general networks are due to the number of patient transfers that could lead to structural differences between the specific patient population flows. We first compared the general patient network to two sets of 1000 networks built from a subset of randomly chosen patients: in the first set we selected the same number of patients as the HAI-specific network (21276 patients) at random and in the second set the same number as in the suspected-HAI network (394859 patients) at random 1000 times and reconstructed each network. Overall, both sets of random patient networks (RP) were smaller in size compared the general network in terms of the number of nodes, edges, edge weight, and as a result average degree (Table 4). In addition, most of the diameters and all average path lengths were larger in the RP networks. The diameters and path lengths of the RP networks are skewed and not normally distributed (p< 0.001, Shapiro-Wilk normality test). As a result, the number of patients used to reconstruct the networks did have an impact of network characteristics.

**Table 4:** Network characteristics of the random patient networks.

Network Topology Measures	General Network (GN)	Suspected-HAI Network (SHAIN)	1000 Suspected-HAI-like RP networks			HAI-Specific Network (HAISN)	1000 HAI-Specific-like RP networks		
			Mean	% ≤ GN	% ≤ SHAIN		Mean	% ≤ GN	% ≤ HAISN
Nodes	2063	1975	2032	100%	0%	1266	1583	100%	0%
Edges	50026	18812	22139	100%	0%	3722	3882	100%	0.3%
Average Edge Weight	14.02	4.92	5.43	100%	0%	2.31	1.62	100%	100%
Average Degree	48.50	19.05	21.79	100%	0%	5.88	4.91	100%	100%
Diameter	30	64	61.59	1.9%	63.0%	47	36.27	7.0%	98.8%
Average Path Length	2.99	3.63	3.78	0%	0%	5.23	8.24	0%	0%
Global Clustering Coefficient	0.23	0.16	0.19	100%	0%	0.08	0.09	100%	2.7%
Density	0.012	0.005	0.005	100%	0%	0.002	0.0016	100%	100%
Average Edge Betweenness	301	852	796	0%	100%	1557	2384	0%	0%
Average Total Closeness	1.6E-4	7.4E-5	1E-3	100%	11.5%	3.2E-5	1.7E-5	100%	100%

Comparison of the healthcare network topology measures with the average measures of 1000 simulated random patient (RP) networks that were composed of the same number of patients as the patient-specific healthcare network. The proportion of network measures equal to and less than the general network and the proportion equal to and less than the patient-specific network measures are shown in percent %. Note: “E” refers to the E-notation for the scientific notation of “×10” followed by the power.

We then compared the characteristics of the HAI-specific and suspected-HAI networks to the RP networks with the same number of patients to assess if HAI patients modified network structure differently than other patients. Overall, the RP networks were larger than their HAI-specific and suspected-HAI healthcare networks analogues meaning that HAI patients were transferred to fewer hospitals than other patients (Table 4). However despite these differences, for some networks measures such as diameter, average path length, and global clustering coefficient, there was less of a difference between the RP networks and the HAI networks than the RP networks and the general network. For example, 63% of suspected-HAI-like RP networks had a diameter equal to or less than that of the suspected-HAI network (64) while 1.9% of these networks had a diameter equal to or less than that of the general network (30). The average diameter (61.59) and the average path lengths (3.78) of these RP networks approached more of that of the suspected-HAI network than the general network. Thus having controlled for the number of patients and thus the size of the network, the differences observed between the suspected-HAI and the general network diameter and average path length may have been due to the suspected-HAI network being a subset of the healthcare network rather than due to differences between HAI patient transfer patterns and non-HAI patient transfers.

## Discussion

In this study we show that the French healthcare networks have heterogeneous patient flows, demonstrate characteristics of small-world and scale-free networks, and are characterized with highly centralized movement of patients towards hub hospital centers. Hub hospitals are characterized as university hospitals and private hospitals in the large metropoles that dominate patient flow. The healthcare networks displayed two-level community clustering: regional community clustering reflecting the French administrative structure, and department or county-level clustering. Certain patient transfer trajectories play a more important role in transferring patients between the regional and departmental communities. Despite differences in the patient population and size, both the HAI-specific and suspected-HAI specific healthcare networks seem to rely on the same underlying structure as that of the general healthcare network.

Due to weak sensitivity and specificity of the PMSI database to detect nosocomial infections with the sole ICD-10 Y95 diagnostic, the HAI-specific network is not reliable in demonstrating the real patient movement patterns for those infected with an HAI.[24–28] There was no confirmation if an infection was absent during admission and if an infection appeared during the first 48 hours of their stay. We suspect that the degree ranking differences and the low percent of acute-care facilities, notably private hospitals, in the HAI-specific network may be due to differences in coding practicing among hospitals rather than the epidemiology of HAIs. The suspected-HAI network reflects a standardized list of diagnoses used by the French HAI surveillance network which has been shown to be more specific and sensitive at detecting patients with HAIs.[28, 29] Having considered the network size differences in the patient-specific networks and the general network, we show that despite the differences in size of the patient population, both the HAI-specific and suspected-HAI specific healthcare networks seem to rely on the same underlying structure as that of the general healthcare network. Indeed, patient-specific networks are a subset of the general patient network and are subject to the same network dynamics.

Public university hospital centers and private hospitals in the main metropoles of France dominate patient flow. A study conducted in the Bourgogne region of France has shown that patient flow was centered towards the university hospital that admitted patients from the entire region and based on the regional proximity of the patients’ residence and patients also sought care in two of the closest main healthcare hubs for specialized care (Paris or Lyon).[30] Highly connected hospitals may harbor more MRSA and MRSA bacteremia cases and may have the most potential to transmit HAIs in the entire network.[12, 13, 31, 32] HAIs may spread at a higher rate than expected at random due to the centralization of patient movement and due to the small average number of transfers required for patients to move throughout the network. A 2012 point prevalence study has shown that HAIs are most prevalent in cancers centers, university hospitals, and armed forces.[33] HAI prevalence was high in the Ile-de-France region which has many hubs, and the north-eastern regions which were not reflected by a higher number of transfers in the patient specific.[33] Albeit some difference in prevalence and patient transfer patterns, hubs should be proposed as targets for sentinel surveillance in addition to priority targets of HAI control strategies where HAI is most prevalent to achieve the most effective reduction in transmission across the country.[15]

Regional community clustering patterns as a form of network connectedness are also important in the development of strategies for coordinated HAI control.[8, 13] Our regional community clustering findings are consistent with that of the healthcare network of England in which communities tend to share more patients among clusters of hospitals in addition to patient flows centered towards a university hospital within the community.[13] Important intermediary trajectories may play a key role in the spread of HAI between hub hospitals and between communities. A study has shown that modifying the number of patients moving between communities may reduce the spread of MRSA.[34] Furthermore, we demonstrated that a two-tier hospital community exists. Depending on the clustering algorithm used, we identified clustering of healthcare communities at the regional level, consistent with the French administrative regions, and department-level communities and inter-departmental hospital clusters that took into account the directionality of patient flow. Coordinated department-level control such as screening of patients based on the identification of key department-level cluster admissions may be the first line of defense against HAI spread within the regions before spread reaches the hub university hospitals. We identified differences between department-level communities of the suspected-HAI and the general network that were overlooked at the regional community level. This may be important in distinguishing hospitals with higher potential to harbor HAI patients, with possible consequences in terms of HAI spread prediction.

Studies have proposed reducing hospital connectedness in order to reduce the risk of epidemic spread of HAI in networks.[13, 35] Decentralization of the healthcare system and more specifically human resource and specialized health services towards the regional and department levels may help reduce the high connectedness of hubs in the metropole centers and redirect patient transfers. France has moved towards regionalization strategies with the creation of regional hospital agencies, albeit not very effective.[36, 37] In addition, the number of university hospitals may be insufficient, below that of the UK, a country with a similar population size. We recommend increasing the number facilities providing specialized services and distributing them at the local level to help redirect patient flow and potentially avoid large-scale HAI dispersal.

We considered temporal dynamics, masked in a network constructed with data for the entire year of 2014, in which observed that monthly healthcare networks were smaller and displayed less centralized patient flow; hence, infection control strategies–for short-term control–should rely more on the local department-level dynamics to minimize hospital-level outbreaks and transmission to neighboring hospitals. In the long term, regional community dynamics may give us clues regarding the gradual propagation of specific HAI pathogens over time assuming HAI carriage patterns follow that of patient flow patterns in the healthcare networks. Further studies are required to assess the temporal dynamics of HAI spread in networks to identify any potential seasonality patterns of flow and how to prevent emerging multi-drug resistant bacteria from becoming endemic.

Our study was subject to certain limitations which should be considered. Many of the university hospitals represent more than one public hospital or healthcare facility due to sharing the same identification number. For example, the largest outlier hub in Paris (AP-HP) represented 39 hospitals, 12 hospitals and 2 specialized health facilities constituted Hospices Civils de Lyon, 9 hospitals make up the university hospital of Toulouse, and 4 hospitals make up the APHM of Marseille. Consequently, university hospital centers accommodated a larger patient population than hospital centers or local hospitals, influencing the network characteristics, which may have led us to overestimate the specific patient movement patterns to and from these centers. However, the high concentration of other hospitals especially hub private hospital centers, armed forces hospitals, cancer centers, psychiatric hospitals, and private post-operative and rehabilitation centers within proximity of these public hospital hubs demonstrates that despite this, major cities such as Paris play the most important role in connecting patients in the national network and that the French healthcare network is a highly centralized system.

The healthcare networks did not include patient flow from private nursing homes that have been shown to play an important role in HAI spread.[38–42] Without private nursing homes included in our study, our results only describe the network topology of hospital patient populations which may be both younger, have shorter duration stay, and may spread HAI differently than the complete nursing home population. As a result, transmission dynamics in our networks may over or underestimate average hospital centrality measures, the volume of patient movements, and the speed at which HAI can spread.

By considering all HAIs as a whole, our networks and recommendations reflect action for a broad spectrum of HAIs; however, one should consider that specific HAIs can vary in terms of carriage and transmission patterns. In addition, recommendations based on our networks would overlook the potential exposure to community-acquired infections, although these may later spread in hospital settings, leading to healthcare-associated outbreaks. Future studies should consider all potential components of patient exposure to both community-associated and healthcare-associated infections and account for individual exposure histories to these infections.

Despite these limitations, our study provides a first description and analysis of the healthcare networks in France. The identified characteristics and community structures may greatly improve future inter-hospital HAI control strategies. The general patient network responds best to informing regional control strategies targeting key patient trajectories and hub hospital centers. We show that the scale-free structure, the number of communities, and their distribution over the country remain qualitatively similar across all networks and that patient-specific networks rely on the underlying structure of the general patient network. Future studies should take into consideration network topology in the prediction of HAI spread and should consider the potential impact of different community definitions for multi-level infection control strategies.

## Methods

### Materials

The Programme de Médicalisation des Systèmes d’information (PMSI) database, a comprehensive French medico-administrative database of hospital activity and patient discharge information, is used to construct the hospital networks.[24, 25] The PMSI database has been used for epidemiological and medical research regarding HAIs.[24–28, 43] A lack of sufficient specificity and sensitivity of the PMSI to detect HAIs is highlighted in these studies. Comparison between laboratory data and hospital data shows that the PMSI has limited coverage of detecting nosocomial conditions.[25–28]

Hence, Gerbier et al. [28] use patient discharge summaries from the PMSI to detect nosocomial infections in the University Hospital of Lyon in 2006 and 2007 for the identification of HAIs in surgery, intensive care and obstetric units. They compare the PMSI data to a gold standard by systematic review of patient files for those classified under surgery, the Centre de Coordination de la Lutte contre les Infections Nosocomiales (CClin) Southwest surveillance network for ICU patients, and a combination of surveillance data from CClin and patient information data for obstetrics. The list of ICD-10 codes related to nosocomial conditions, which we entitle “suspected-HAIs,” can be found in S1 Annex. Gerbier et al. find a sensitivity and specificity for case identification of nosocomial infections to be 26.3% (95% CI 13.2–42.1) and 99.5% (95% 98.8–100.0) for the identification of surgical site infections (78.9% and 65.7% by expanding the number of diagnostic codes) respectively; 48.8% (95% CI 42.6–55.0) and 78.4% (95% CI 76.1–80.1) in intensive care respectively, and 42.9% (95% CI 25.0–60.7) and 87.3% (95% CI 85.2–89.3) for identification of postpartum infections respectively.[28]

### Inclusion and exclusion criteria

Using patient transfer data from 2014, three healthcare networks are reconstructed based on the following criteria:

■ All patient transfers (non-specific diagnoses)
■ Patient transfers with the ICD-10 code of Y95 (for nosocomial conditions or HAI) as their principal, related, or associated diagnosis in the medical, surgery, obstetric hospitals (MCO) and postoperative and rehabilitation centers (SSR)
■ Patient transfers identified with all possible and suspected cases of HAIs in the surgical, intensive care, and obstetric wards in 2014, by referencing the diagnoses with known specificities and sensitivities listed in Gerbier et al. publication[28] with supplementary information from other publications.[24–26]

Only direct transfers of patients who are discharged from a hospital and sent to another in another jurisdiction (“transfer”) or those who are discharged from one medical unit and move to another in the same hospital jurisdiction (“mutation”) are included. The hospital discharge summaries reflected the overall hospital stay of patients and a single diagnosis made them eligible without specification if it occurred during admission or at discharge. Patients who are discharged to their residence or deceased in a hospital are excluded. Patients hospitalized in non-continental European departments are also excluded.

### Construction of patient transfer network matrices

First, the networks of hospitals and healthcare centers are re-built *in silico* using patient transfer data to model the potential movement of patients with HAIs from one hospital to another. In the PMSI database, each patient discharge summary contains information on the hospital facility of stay. Each hospital facility is identified by its unique FINESS number (Fichier National des Etablissements Sanitaires et Sociaux).[44] For this study, the FINESS and stay number of each patient discharge summary are used to merge two PMSI databases: one for acute-care and one for long-term care hospitals. Each patient stay is also numbered by order of stay across different hospitals. To create the logical sequence of patient movement, we sort each discharge summary: by patient ID and patient stay number for all observations.

The adjacency matrix [21], a graph of N nodes and E edges can be described by its’ *N* × *N* adjacency matrix A defined as:

$$A_{ij}=\left\{ \begin{array}{l} \;=1\;\mathrm{if}\;i\;\mathrm{and}\;j\;\mathrm{are}\;\mathrm{connected}\\ \;=0\;\mathrm{otherwise} \end{array}\right.$$

In our patient transfer network, nodes (N) are defined as hospitals and edges (E) as the patient trajectories that connect hospitals. We computed origin *i* and target *j* hospitals for each patient stay by assessing if for each discharge the patient entered the hospital *i* as a transfer or mutation and left hospital *i* as a transfer or mutation. The same is computed for each *j* hospital. Using the iGraph package for R statistical software, we create the adjacency matrix of all *i* and *j* hospitals, including *i* and *j* if *i* did not transfer out any patients but received them and vice versa for *j*.[45]

We also compute the number of patients moving between hospitals *i* and *j*, as w_*ij*_. The sum of the edge weights of the adjacent edges, the weight strength, is given by:

$$s_{i}^{w}=\sum_{j\in \Gamma (i)}w_{ij}$$

in which Γ(*i*) is the set of neighbor hospitals of *i*.[21] Edge weights represent the number of patients within the trajectories between two healthcare facilities.

To identify the most important hospitals of a network, a series of centrality measures are calculated. The degree of a hospital, *k*, is the number of hospitals one hospital is connected to through its patient trajectories [21] defined as:

$$k_{i}=\sum_{j}A_{ij}$$

The average degree of a network[21] is given by:

$$\langle k\rangle =\frac{1}{N}\sum_{i}k_{i}=\frac{2E}{N}$$

In addition, A_*ij*_ is a directed graph in which the directionality of patient transfers from one hospital to another is taken into account. Consequently, we can calculate the indegree (deg^-^) and outdegree (deg^+^) of any given node in which the degree sum formula is given by:

$$\sum_{n\in N}{deg}^{+}(n)=\sum_{n\in N}{deg}^{-}(n)=|E|$$

Betweenness centrality measures the importance of hospital acting as an intermediary between other hospitals defined as:

$$g(i)=\sum_{s\neq t}\frac{\sigma_{st}(i)}{\sigma_{st}}$$

Where betweenness centrality *g*(*i*) is equal to the sum of the σ_*st*_ the number of shortest paths going from *s* to *t* through hospital *i* measuring the importance of hospital *i* to the organization of flow in the network.[21] The same measure is calculated for patient trajectories defined as:

$$g(e)=\sum_{e\in E}\frac{\sigma_{st}(e)}{\sigma_{st}}$$

where edge betweenness centrality *g*(*e*) is equal to the sum of the σ_*st*_ the number of shortest paths going from s to *t* through edge *e* measuring the importance of edge *e* to the organization of flow in the network.[21]

### Community clustering

Two community detection algorithms are used to assess community clustering for each network, which both take into account weighted graphs.[45] A common measure of the quality of partitions of a network into communities of densely connected nodes is modularity. Modularity is a scalar value between the vales of -1 and 1 that measures the density of links inside communities compared to links between them.[21, 22] The modularity and different communities of our network are defined using a community detection algorithm. The Greedy algorithm developed by Clauset et al.[19] optimizes modularity as the algorithm relies on network formation and as a result, computes a smaller range of communities as modularity approaches 1; however, the Greedy algorithm does not take into account edge directionality and we detect communities for undirected graphs of the healthcare networks. On the other hand, the Map equation algorithm developed by Rosvall et al. detects communities based on patterns of flow and takes into account edge directionality and the directed graphs are assessed.[20] This algorithm detects communities based on network structure and how it influences the system’s behavior.

Based on the community partitioning for each network, the mean geographic distance between hospitals of the same community is measured. To geo-localize hospitals, we used public government data on French hospital facilities and postal code addresses (https://www.data.gouv.fr/). Using an online batch geocoding server (http://www.findlatitudeandlongitude.com/), the hospitals’ addresses were converted to latitude and longitude coordinates. A distance matrix was calculated using the haversine formula to measure great-circle distances between all hospitals.[46]

Two intercommunity matrices were developed to assess patient sharing between different communities 1) Greedy algorithm-based communities 2) Map Equation-based communities. Based on the algorithm, each hospital node is assigned a community number. A matrix summing the individual hospitals transfers for hospitals that share the same community is constructed and converted into a directed graph. In addition, the mean latitude and longitude are calculated for each community from individual geocodes of the member hospitals. For the Map Equation intercommunity network, the Greedy algorithm is applied to identify the number of communities present when modularity is maximized.

### Ranking of hospitals

Hospitals were ranked by their degree, betweenness, and closeness centrality measures for each network. When the centrality measures were equal, we replaced the rankings by the mean rankings. We tested the differences between rankings on an increasing subset of shared hospitals with the Wilcoxon rank sum test. The test was conducted as follows: starting with the highest ranked hospital in the general network, adding the next ranked general network hospital, and testing for significant differences between the general network rank and either the HAI-specific or suspected-HAI network rank of the same hospital until we compared all shared hospitals. As a result, we determined the thresholds where hospital rankings across the networks start to significantly differ which was defined as Wilcoxon rank sum test p-values under the 5% alpha risk.

### Random patient networks

To compare the networks between each other, we built 1000 random patients networks from the general network. We selected the same number of patients as either the HAI (21276 patients) or suspected HAI networks (394859 patients) from the general patient network at random and reconstructed these networks using their hospital discharge summaries. We calculated various network measures and the proportion of random patient networks that had values greater than, equal to, or less than the general patient network and the respective patient-specific networks.

## Supporting information

S1 Annex All transfer patients considered as suspected to have a hospital-acquired infection. (PDF)

S1 Text Power-law behavior: average strength s(k) as a function of degree k. (PDF)

S2 Text Power-law, log-normal, and Poisson distribution goodness-of-fit tests (PDF)

S3 Text “Small-world” network characteristics (PDF)

S4 Text Comparison with Erdos-Renyi random networks. (PDF)

S5 Text How do the communities vary across the networks? (PDF)

S6 Text Does the general healthcare network change with the age of the patients? (PDF)

S7 Text What are the temporal dynamics of the general healthcare network?. (PDF)

S1 Fig Average strength and degree distribution of the general network. The degree k represents the number of hospital connections of each hospital in the general network and the average strength s(k) stands for the number of patient transfers as a function of degree. The number of patient transfers and number of hospital connections were highly positively correlated (r = 0.91). The best-fitting power law model was s(k) = k1.51 (dashed line). The curves for s(k) = k (dotted line) and s(k) = 10*k (dash-dot line) are shown for comparison.
(PDF)

S2 Fig Average strength and degree distribution of the suspected-HAI network. Distribution of hospital connections k of each hospital in the suspected-HAI network and the average strength s(k) or number of patient transfers as a function of degree. The number of patient transfers and number of hospital connections were highly positively correlated (r = 0.95). The best-fitting power law model was s(k) = k1.36 (dashed line). The curves for s(k) = k (dotted line) and s(k) = 10*k (dash-dot line) are shown for comparison.
(PDF)

S3 Fig Average strength and degree distribution of the HAI-specific network. Distribution of hospital connections k of each hospital in the HAI-specific network and the average strength s(k) or number of patient transfers as a function of degree. The number of patient transfers and number of hospital connections were highly positively correlated (r = 0.99). The best-fitting power law model was s(k) = k1.26 (dashed line). The curves for s(k) = k (dotted line) and s(k) = 10*k (dash-dot line) are shown for comparison.
(PDF)

S4 Fig Cumulative distribution functions and fit for degree and strength distribution of the general, suspected-HAI, and HAI-specific network. Cumulative distribution functions of degree k (top left) and strength s (bottom left) for the general network, suspected-HAI network (top center, bottom center), and HAI-specific network (top right, bottom right). Fitted power-law (red), log-normal (green), and Poisson (blue) distributions are shown when: x-min for degree = 77 and strength = 1191 in the general network; x-min for degree = 20 and strength = 119 in the suspected-HAI network; and x-min for degree = 7 and strength = 32 in the HAI-specific network.
(PDF)

S5 Fig Cumulative distribution functions and fit for indegree and instrength distribution of the general, suspected-HAI, and HAI-specific network. The cumulative distribution functions of k- indegree for the general network (top left) and s- instrength (bottom left), suspected-HAI networks (top center, bottom center), and HAI-specific network (top right, bottom right). Fitted power-law (red), log-normal (green), and Poisson (blue) distributions are shown when: x-min for indegree = 36 and instrength = 698 in the general network; x-min for indegree = 13 and instrength = 131 in the suspected-HAI network; and x-min for indegree = 5 and instrength = 18 in the HAI-specific network. Power-law and log-normal had good fit for indegree and instrength in the three networks (KS-statistic p-values > 0.15) with the exception of log-normal distribution of indegree in the general and suspected-HAI network (KS-statistic p-value < 0.04). Poisson distribution was not a good fit for indegree and instrength in all networks (KS-statistic p-value < 0.0001).
(PDF)

S6 Fig Cumulative distribution functions and fit for outdegree and outstrength distribution of the general, suspected-HAI, and HAI-specific network. The cumulative distribution functions of k+ outdegree for the general network (top left) and s+ outstrength (bottom left), suspected-HAI networks (top center, bottom center), and HAI-specific network (top right, bottom right). Fitted power-law (red), log-normal (green), and Poisson (blue) distributions are shown when: x-min for outdegree = 101 and outstrength = 1102 in the general network; x-min for outdegree = 27 and outstrength = 70 in the suspected-HAI network; and x-min for outdegree = 7 and outstrength = 3 in the HAI-specific network. Only power-law distribution had a good fit for both outdegree and outstrength (KS-statistic p-values > 0.41) while log-normal distribution was only a good fit for the HAI-specific network (KS-statistic p-value = 0.15).
(PDF)

S7 Fig Shortest path length distributions in the networks. **The** length of the shortest paths or steps between any two nodes in the networks are calculated and plotted by their frequency.
(PDF)

S8 Fig Distributions of p-values of hospital rank subsets using the Wilcoxon rank sum test in the HAI-specific network compared to the general network hospital ranks. (PDF)

S9 Fig Distributions of p-values of hospital rank subsets using the Wilcoxon rank sum test in the suspected-HAI network compared to the general network hospital ranks. (PDF)

S10 Fig Healthcare networks by age for all patients. Healthcare networks for all transferred patients (a) aged 18 and younger (b) aged 18 to 60 (c) older than 60 years old. Greedy communities were colored by the corresponding general network community regions with additional community color if not present for (a) 23 communities in which 3 were black (<5 hospitals) (b) 17 communities in which 1 was black (<5 hospitals) (c) 29 communities in which 14 were black (<5 hospitals).
(PDF)

S11 Fig Healthcare networks by age for suspected-HAI patients. Healthcare networks for all transferred patients (a) aged 18 and younger (b) aged 18 to 60 (c) older than 60 years old. We detected a total number of Greedy-based communities for each age network (a) 22 total and 19 with over 2 hospitals from a network of 1894 hospitals and 11234 edges (b) 30 total with 17 with over 2 hospitals from a network of 1559 hospitals and 5423 edges (c) 29 total with 14 with over 2 hospitals per community from a network of 218 hospitals and 318 trajectories.
(PDF)

S12 Fig Healthcare networks by age for HAI-specific patients. Healthcare networks for all transferred patients (a) aged 18 and younger (b) aged 18 to 60 (c) older than 60 years old. Network communities are detected using the Greedy algorithm and colored according to community membership for (a) 1143 hospitals and 2260 edges with 50 total communities with only 28 composed of more than 5 hospitals (b) 603 hospitals and 593 edges with 41 total communities and 19 with over 5 hospitals (c) 44 hospitals and 33 edges, 18 total communities, 2 communities with more than 5 hospitals, and 9 communities with more than 2 hospitals.
(PDF)

S1 Table Network characteristics of the Erdos-Renyi random networks. Comparison of the healthcare network topology measures with the average measures of 100 simulated Erdos-Renyi (ER) networks that are parameterized with same number of nodes, edges, and Poisson-distributed average edge weight. For each measure, a t-test is conducted to compare the difference between the health network value and the average values of the ER networks with given 95% confidence intervals and p-values.
(PDF)
