# Supplementary material for: Spread of hospital-acquired infections: A comparison of healthcare networks
Source: PLoS Comput Biol. 2017 Aug 24;13(8):e1005666. doi: 10.1371/journal.pcbi.1005666 (PMC5570216; doi:10.1371/journal.pcbi.1005666)
Supplement: S1 Annex — (PDF) [file pcbi.1005666.s001.pdf]

S1 Annex. All Transfer Patients Considered as Suspected to have a Hospital-Acquired Infection

Suspected-HAI patients are identified by the presence of at least one of the following International Classification of Diseases, ICD-10 codes at the principal, related, or associated diagnosis surveyed by the PMSI database [24, 28]:

Nosocomial condition: Y95

Surgical site infection: T814, T815, T816, T826, T827, T835, T836, T845, T846, T847, T857, O860

Extensive infection: T813, T818, T888, T889, K316, K603, K604, K605, K632, K823, K833, N360, N823, Z090, Z094, Z097, Z098, Z099, Z480, Z488, Z489, R50, R500, R501, R09, A40, A400, A401, A402, A403, A408, A409, A41, A410, A411, A412, A413, A414, A415, A418, A419, A427, T874

Pneumonia: J10-, J11-, J12-, J13-, J14-, J15-, J16-, J17-, J18-

Urinary infection: N300, N34-, N390, O862, O863, T835

Bacteremia: A021, A207, A217, A227, A241, A267, A280, A327, A392, A393, A394, A40-, A41-, A427, A483, A499, A548, B007, B377, O080, O753, O85, P3600, P3610, P3620, P3630, P3640, P3650, P3680, P3690

Endometritis: N710, N719, N72, O235, O85

Breast infection: O91-

Uncategorized infections: O861, O864, O868
