# Supplementary material for: Spread of hospital-acquired infections: A comparison of healthcare networks
Source: PLoS Comput Biol. 2017 Aug 24;13(8):e1005666. doi: 10.1371/journal.pcbi.1005666 (PMC5570216; doi:10.1371/journal.pcbi.1005666)
Supplement: S1 Text — (PDF) [file pcbi.1005666.s002.pdf]

S1 Text. Power-Law Behavior: Average Strength s(k) as a Function of Degree k

To better understand the heavy tailed behavior in the networks, we plotted average patient transfers and hospital connectedness or degree using the following formula given by Barrat et al.[1]:

$$s(k) \sim k^{\beta}$$

The general healthcare network's average strength given as a function of degree *k, s(k),* varied with a power β of 1.51 (S1 Fig). For the suspected-HAI networks and the HAI-specific network average strength varies by a power of 1.36 and 1.26 as a function of degree respectively (S2 Fig, S3 Fig). Therefore, in these healthcare networks, the number of patients transferred by a hospital increased at a higher rate than that of the hospital's connections and was most high in the general network.

[1] Barrat A, Barthelemy M, Pastor-Satorras R, Vespignani A. The architecture of complex weighted networks. Proc Natl Acad Sci U S A. 2004;101(11):3747-52. doi: 10.1073/pnas.0400087101. PubMed PMID: 15007165; PubMed Central PMCID: PMCPMC374315.
