# Supplementary material for: Spread of hospital-acquired infections: A comparison of healthcare networks
Source: PLoS Comput Biol. 2017 Aug 24;13(8):e1005666. doi: 10.1371/journal.pcbi.1005666 (PMC5570216; doi:10.1371/journal.pcbi.1005666)
Supplement: S2 Text — (PDF) [file pcbi.1005666.s003.pdf]

S2 Text. Power-Law, Log-Normal, and Poisson Distribution Goodness-Of-Fit Tests

Power law distribution of degree is a key characteristic of "scale-free" networks, in which a small number of nodes are highly connected.[1,2] We described the distribution of the degree and strength of our directed healthcare networks and tested the fit of the (1) power-law, (2) log-normal, and (3) Poisson distributions to the data (S4 Fig). Using a goodness of fit test via a bootstrapping procedure, we found that the power law distributions had good fit for degree in all three healthcare networks (Kolmogorov-Smirnoff (KS) statistic p-values > 0.07) evidencing that they indeed displayed scale-free characteristics. The power law distributions for strength had good fit in all three networks as well (KS statistic p-values > 0.2). Log-normal distribution was also a good fit for degree in the three networks (KS statistic p-values > 0.27), as well as for strength in the general and HAI-specific networks, but not in the suspected-HAI network (KS statistic p-values = 0.02). Finally, Poisson distribution was not a good fit for either degree or strength in all three networks, demonstrating that the healthcare networks were heterogeneously distributed (KS statistic p-values < 0.001 for all).

Goodness of fit tests were also performed for distributions for k- indegree, k+ outdegree, s- instrength and s+ outstrength, showing similar results (S5 Fig, S6 Fig). In addition, the average strength as a function of degree also exhibited a power-law behavior, with higher power in the general healthcare network, followed by the suspected-HAI network, and HAI-specific network (S1 Text, S1 Fig, S2 Fig, and S3 Fig). Therefore, all three healthcare networks displayed scale-free properties with a limited number of highly connected "hub" hospitals.

---

[1] Barrat A, Barthelemy M, Pastor-Satorras R, Vespignani A. The architecture of complex weighted networks. Proc Natl Acad Sci U S A. 2004;101(11):3747-52. doi: 10.1073/pnas.0400087101. PubMed PMID: 15007165; PubMed Central PMCID: PMCPMC374315.
[2] Barabási A-L, Albert R. Emergence of Scaling in Random Networks. 1999. doi: 10.1126/science.286.5439.509.
