# Supplementary material for: Spread of hospital-acquired infections: A comparison of healthcare networks
Source: PLoS Comput Biol. 2017 Aug 24;13(8):e1005666. doi: 10.1371/journal.pcbi.1005666 (PMC5570216; doi:10.1371/journal.pcbi.1005666)
Supplement: S3 Text — (PDF) [file pcbi.1005666.s004.pdf]

S3 Text. "Small-world" network characteristics

In addition to assessing the scale-free properties of the networks, we assessed the "small-world" characteristics to determine if nodes were closely within reach of all others in the network. Small average path length and large clustering coefficients are indicators of a small-world effect.[1] All three healthcare networks had smaller diameters and smaller average path length given their size compared to Erdos-Renyi random networks (S4 Text, S1 Table), indicating that hospital subpopulations were within close topological proximity to one another and that patients, once admitted to any hospitals, could be more easily sent to all hospitals in the network within a few number of transfers (Table 1). The largest network in size, the general patient network, had a diameter of 30, defined as the longest of the shortest distance between any two nodes in the network, and an average path length of 2.99, given by the average shortest path between all possible pairs of connected nodes in the network. In S7 Fig, the distribution of the shortest path lengths across the networks is shown. The general network has a higher frequency of path lengths between zero and five whereas in the HAI-specific networks, the frequency is reduced and the longer path lengths become more frequent. Therefore, hospitals within the general network were more efficient in transfer patients.

Further analysis of the networks also supported this observed small-world characteristic. Graph density, as observed in the three networks, is the total proportion of existing edges out of the potential edges that can exist to connect all nodes together. Computation of densities indicated that only 0.2%, 0.5%, and 1.2% out of all possible connections exist in the HAI-specific, suspected-HAI, and general patient networks respectively (Table 1). Hospitals shared patients with a limited number of other hospitals in the network. Moreover, the global clustering coefficient (GCC), which gives an overall indication of the clustering or number of triangles

[1] Watts DJ, Strogatz S, H. Collective dynamics of 'small-world' networks. Nature. 1998;393(6684):440-2. doi:10.1038/30918.

(triplets of nodes) existing among the possible connected ones,[2] was high in the three networks compared to a random network of the same size (S4 Text, S2 Table), especially in the general healthcare network. Therefore, hospitals sending patients to the same hospitals were more likely to be linked together by patient sharing.

[2] van der Hofstad R. Random Graphs and Complex Networks. Eindhoven: Cambridge University Press; 2016.
