# Supplementary material for: Spread of hospital-acquired infections: A comparison of healthcare networks
Source: PLoS Comput Biol. 2017 Aug 24;13(8):e1005666. doi: 10.1371/journal.pcbi.1005666 (PMC5570216; doi:10.1371/journal.pcbi.1005666)
Supplement: S4 Text — (PDF) [file pcbi.1005666.s005.pdf]

S4 Text. Comparison with Erdos-Renyi Random Networks

Given that the healthcare networks differ in size, they cannot be compared directly for all network properties. To further assess the distinct properties of the healthcare networks, we compared each of the three healthcare networks to 100 simulated Erdos-Renyi (ER) random directed networks parameterized with the same number of nodes, edges, and average edge weight. We constructed 100 Erdos and Renyi random networks[1] with the same number of nodes, edges, and a Poisson distributed edge weight corresponding to total transfers of the three networks. In the Erdos-Renyi model, the fixed number of nodes have the same probability of being connected by a fixed number of edges.[8]

As expected, the random networks displayed less small-world characteristics compared to the healthcare networks. Overall, healthcare networks were more clustered than ER networks (S1 Table). Diameter was smaller in the general healthcare network and larger for the HAI-specific and suspected-HAI networks compared to the average ER network. Average path length was larger in the general healthcare network and suspected-HAI network and smaller in the HAI-specific network indicating that in the HAI-specific network have a closer average distance between any two nodes than that of an ER network. The larger path length in the two largest healthcare networks compared to ER networks may be due to the more heterogeneous distribution of path lengths in the healthcare networks where the distribution of path lengths may vary between highly connected and highly disconnected hospitals. The average total closeness was much smaller in the suspected-HAI network and general network random networks, indicating that hospitals in these networks will be able to disperse their patients in the network quicker, while in the HAI-specific network, patient movement was slower than in an average ER network (S1 Table).

---

[1] Erdos P, Renyi A. On random graphs. Publicationes Mathematicae. 1959;6:290-7.

Heterogeneity in patient trajectories and community clustering also distinguish the healthcare networks and ER networks. Another possible contribution to efficiency of patient movement in the general healthcare network may be due to higher average edge betweenness (301) and a higher maximum edge betweenness (1175) compared to ER networks (252.81 95% CI [252.75-252.88] and 32.02 95% CI [31.74-32.30] in the ER networks respectively). While compared to the average ER network, the average edge betweenness in the HAI-specific and suspected-HAI healthcare networks were lower, maximum edge betweenness of the HAI-specific (73) and suspected-HAI healthcare networks (314) were higher, demonstrating that flow was concentrated in a small number of edges and less evenly distributed in these healthcare networks (S1 Table). ER networks display a smaller number of communities. Hospitals within the same community were geographically further away from one another on average than in the healthcare networks (with the exception of Map Equation-detected communities in the suspected-HAI network). In résumé, the healthcare networks had more centralized and efficient patient flow concentrated in a small number of nodes and edges while the general patient network was more clustered and efficient compared to both the smaller healthcare networks and ER networks.
