# Supplementary material for: Spread of hospital-acquired infections: A comparison of healthcare networks
Source: PLoS Comput Biol. 2017 Aug 24;13(8):e1005666. doi: 10.1371/journal.pcbi.1005666 (PMC5570216; doi:10.1371/journal.pcbi.1005666)
Supplement: S5 Text — (PDF) [file pcbi.1005666.s006.pdf]

S5 Text. How do the communities vary across the networks?

Healthcare networks displayed differences in community clustering of their hospitals which could be important to better understanding activity in the national healthcare system. Visually, community clustering was similar across the three networks (Fig 2). However, on average, each of the 18 Greedy-based community nodes in the general network (Fig 2a) had hospitals belonging to 4.11 different HAI-specific Greedy-based communities (Fig 2c). These 4.11 communities were not evenly distributed and most hospitals in a general network community belonged to only one dominating HAI-specific community. The dominating HAI-specific communities made up for 78% of the general network community hospital composition for shared hospitals between the two networks. Similarly, on average one general network community was made up of hospitals from 2.56 different suspected-HAI communities (Fig 2b). In addition, one suspected-HAI community made up most (92% of the shared hospitals on average) of the general network community composition. As a result, HAI-specific and suspected-HAI healthcare network hospitals shared most of the community composition found in the general network. When taking into consideration only communities containing at least 2 hospitals, the suspected-HAI network had an equal number of communities as the general network for both algorithms (18 Greedy-based communities and 112 Map Equation-based communities). Therefore, the suspected-HAI healthcare network had very similar community patient sharing structure compared to the general network for both algorithms while the HAI-specific network less so, demonstrating that hospitals transfer HAI-specific patients differently than other patients.
