# Supplementary material for: Spread of hospital-acquired infections: A comparison of healthcare networks
Source: PLoS Comput Biol. 2017 Aug 24;13(8):e1005666. doi: 10.1371/journal.pcbi.1005666 (PMC5570216; doi:10.1371/journal.pcbi.1005666)
Supplement: S6 Text — (PDF) [file pcbi.1005666.s007.pdf]

S6 Text. Does the General Healthcare Network Change with the Age of the Patients?

To better categorize the patient profiles in the healthcare networks, we assessed the age distributions of patients since different age populations may display varied pathologies, vulnerabilities, and risks for HAI transmission. Patients across the networks were characterized by older age with an average age for all transferred patients of 61.18 (SD=22.09), an average age of 55.25 (SD=21.65) for patients suspected to have an HAI, and an average age of 64.94 (SD=18.51) for patients diagnosed with an HAI. Women tended to be slightly older than men in the general and HAI-specific networks ($p < 0.05$, t-test). The oldest patient population was in the postoperative and rehabilitation centers for all transferred patients and those suspected to have an HAI (average of 68.60 (SD=18.35) and 67.45 (SD=17.40) respectively).

To assess potential changes in community clustering due to age groups, we reconstructed 3 age-specific networks (1) less than or equal to 18 years old (2) between 18 and 60 years old (3) over 60 years old. Not surprisingly, we found that the largest network was of transfer patients over the age of 60. Results are shown for all transferred patients in S7 Fig and for HAI and suspected-HAI patients in S8 Fig and S9 Fig respectively. As expected, patients over the age of 60 formed a similar network to that of all patient transfers with 1996 nodes, 31427 trajectories, and 23 Greedy communities of which only 21 had more than 5 hospitals (S7a Fig). The middle-aged network was second largest with 1985 nodes, 18304 trajectories, and 17 communities of which 16 had more than 5 hospitals (S7b Fig). On the other hand, children and adolescents consistently made up smaller networks and for all transferred patients of this age group, they created a network of 575 nodes, 1349 trajectories, and 29 communities of which 15 had more than 5 hospitals (S7c Fig). The Greedy-based communities displayed regional geographic clustering of hospitals, differed between age groups, and differed from the general healthcare network. Most importantly, we saw an introduction of three new regional communities in the oldest age group not previously identified (S7a Fig).

To better understand age distribution, which may also play a role in patient movement patterns, we compare the networks obtained for different patient age groups. Although the network is small, we identified that adolescent patient movement is different than that of older patients in terms of relative community size for the largest communities. We identify three new communities unique to patients over the age of 60 that displays some difference in community composition than the general network, which may interest decision makers targeting elderly populations.
