# Supplementary material for: Spread of hospital-acquired infections: A comparison of healthcare networks
Source: PLoS Comput Biol. 2017 Aug 24;13(8):e1005666. doi: 10.1371/journal.pcbi.1005666 (PMC5570216; doi:10.1371/journal.pcbi.1005666)
Supplement: S7 Text — (PDF) [file pcbi.1005666.s008.pdf]

S7 Text. What are the Temporal Dynamics of the General Healthcare Network?

To investigate any existing temporal dynamics of patient admissions, we assessed the number of admissions by month. Patient admissions per month remained stable over the year with a small drop in August during the holidays with an overall monthly average of 979,142 admissions.

We assessed and compared the monthly healthcare networks from the general patient population in order to identify how the healthcare networks changed in size and how patterns of patient flows compared over time. On average, monthly networks were composed of fewer hospitals than the cumulative yearly network (2063 hospitals compared to an average 1218 hospitals 95% CI [1080-1357], p-value < 0.001, t-test). As a result, the number of hospitals connections and the number of patients moving between them was reduced over monthly intervals. We also observed that these networks were slightly less clustered (0.23 GCC in the cumulative network versus an average 0.17 GCC 95% CI [0.17-0.18], p-value < 0.001, t-test) with a larger diameter (30 in the cumulative network versus an average 45.83 95% CI [40.74-50.93], p-value < 0.001, t-test) and path length (2.99 in the cumulative network versus an average 4.86 95% CI [4.77-4.95], p-value < 0.001, t-test) per month on average. Regarding Map Equation communities, monthly networks on average had a smaller number of communities including when considering only communities with more than one hospital (p < 0.001, t-test). On the other hand, there was a larger number of Greedy communities in the monthly networks overall but no difference in number and localization of communities when considering only communities with more than one hospital. Monthly communities may be less clustered and patients may not visit all of the hospitals each month but they still retained the same regional patient sharing patterns seen in the annual network.
