# Supplementary material for: Spread of hospital-acquired infections: A comparison of healthcare networks
Source: PLoS Comput Biol. 2017 Aug 24;13(8):e1005666. doi: 10.1371/journal.pcbi.1005666 (PMC5570216; doi:10.1371/journal.pcbi.1005666)
Supplement: S1 Fig — The degree k represents the number of hospital connections of each hospital in the general network and the average strength s(k) stands for the number of patient transfers as a function of degree. The number of patient transfers and number of hospital connections were highly positively correlated (r = 0.91). The best-fitting power law model was s(k) = k1.51 (dashed line). The curves for s(k) = k (dotted line) and s(k) = 10*k (dash-dot line) are shown for comparison. (PDF) [file pcbi.1005666.s009.pdf]

***S1 Fig. Average Strength and Degree Distribution of the General Network***

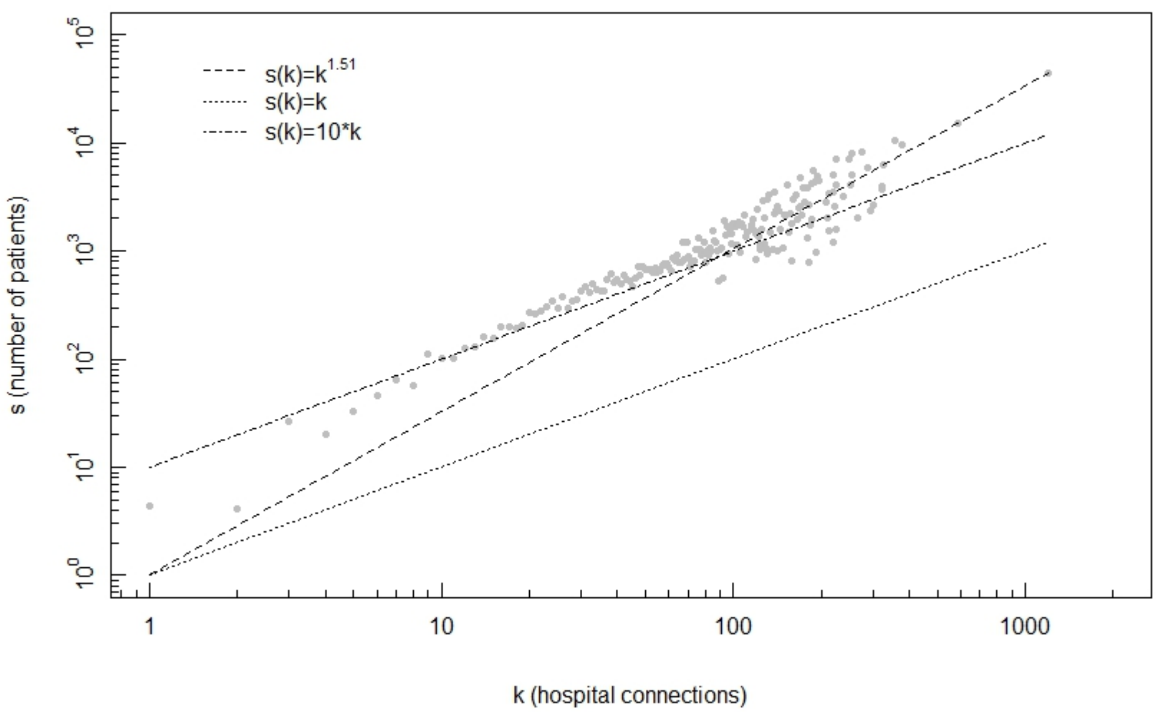


**S1 Fig.** The degree k represents the number of hospital connections of each hospital in the general network and the average strength s(k) stands for the number of patient transfers as a function of degree. The number of patient transfers and number of hospital connections were highly positively correlated (r = 0.91). The best-fitting power law model was $s(k)=k^{1.51}$ (dashed line). The curves for s(k)=k (dotted line) and s(k)=10*k (dash-dot line) are shown for comparison.
