# Supplementary material for: Spread of hospital-acquired infections: A comparison of healthcare networks
Source: PLoS Comput Biol. 2017 Aug 24;13(8):e1005666. doi: 10.1371/journal.pcbi.1005666 (PMC5570216; doi:10.1371/journal.pcbi.1005666)
Supplement: S3 Fig — Distribution of hospital connections k of each hospital in the HAI-specific network and the average strength s(k) or number of patient transfers as a function of degree. The number of patient transfers and number of hospital connections were highly positively correlated (r = 0.99). The best-fitting power law model was s(k) = k1.26 (dashed line). The curves for s(k) = k (dotted line) and s(k) = 10*k (dash-dot line) are shown for comparison. (PDF) [file pcbi.1005666.s011.pdf]

S3 Fig. Average Strength and Degree Distribution of the HAI-Specific Network

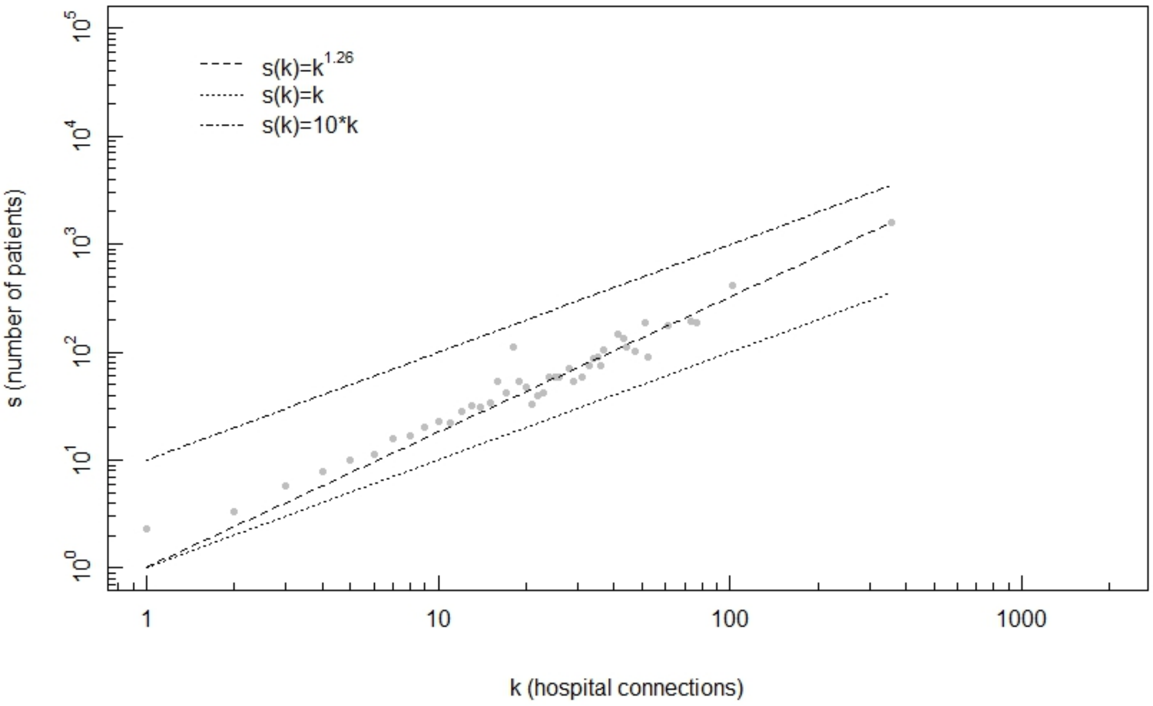

S3 Fig. Distribution of hospital connections *k* of each hospital in the HAI-specifc network and the average strength *s(k)* or number of patient transfers as a function of degree. The number of patient transfers and number of hospital connections were highly positively correlated (r = 0.99). The best-fitting power law model was $s(k)=k^{1.26}$ (dashed line). The curves for s(k)=k (dotted line) and s(k)=10*k (dash-dot line) are shown for comparison.
