# Supplementary material for: Spread of hospital-acquired infections: A comparison of healthcare networks
Source: PLoS Comput Biol. 2017 Aug 24;13(8):e1005666. doi: 10.1371/journal.pcbi.1005666 (PMC5570216; doi:10.1371/journal.pcbi.1005666)
Supplement: S4 Fig — Cumulative distribution functions of degree k (top left) and strength s (bottom left) for the general network, suspected-HAI network (top center, bottom center), and HAI-specific network (top right, bottom right). Fitted power-law (red), log-normal (green), and Poisson (blue) distributions are shown when: x-min for degree = 77 and strength = 1191 in the general network; x-min for degree = 20 and strength = 119 in the suspected-HAI network; and x-min for degree = 7 and strength = 32 in the HAI-specific network. (PDF) [file pcbi.1005666.s012.pdf]

S4 Fig. Cumulative Distribution Functions and Fit for Degree and Strength Distribution of the General, Suspected-HAI, and HAI-Specific Network

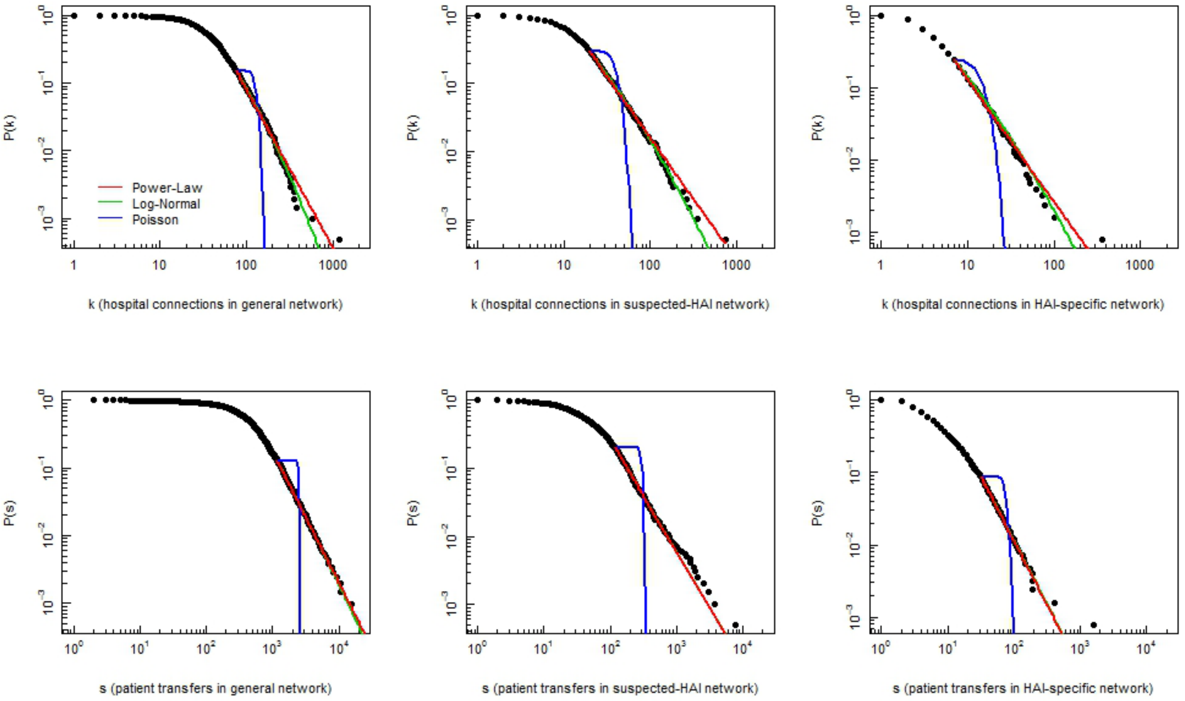

S4 Fig. Cumulative distribution functions of degree *k* (top left) and strength *s* (bottom left) for the general network, suspected-HAI network (top center, bottom center), and HAI-specific network (top right, bottom right). Fitted power-law (red), log-normal (green), and Poisson (blue) distributions are shown when: x-min for degree = 77 and strength = 1191 in the general network; x-min for degree = 20 and strength = 119 in the suspected-HAI network; and x-min for degree = 7 and strength = 32 in the HAI-specific network.
