# Supplementary material for: Spread of hospital-acquired infections: A comparison of healthcare networks
Source: PLoS Comput Biol. 2017 Aug 24;13(8):e1005666. doi: 10.1371/journal.pcbi.1005666 (PMC5570216; doi:10.1371/journal.pcbi.1005666)
Supplement: S5 Fig — The cumulative distribution functions of k- indegree for the general network (top left) and s- instrength (bottom left), suspected-HAI networks (top center, bottom center), and HAI-specific network (top right, bottom right). Fitted power-law (red), log-normal (green), and Poisson (blue) distributions are shown when: x-min for indegree = 36 and instrength = 698 in the general network; x-min for indegree = 13 and instrength = 131 in the suspected-HAI network; and x-min for indegree = 5 and instrength = 18 in the HAI-specific network. Power-law and log-normal had good fit for indegree and instrength in the three networks (KS-statistic p-values > 0.15) with the exception of log-normal distribution of indegree in the general and suspected-HAI network (KS-statistic p-value < 0.04). Poisson distribution was not a good fit for indegree and instrength in all networks (KS-statistic p-value < 0.0001). (PDF) [file pcbi.1005666.s013.pdf]

S5 Fig. Cumulative Distribution Functions and Fit for Indegree and Instrength Distribution of the General, Suspected-HAI, and HAI-Specific Network

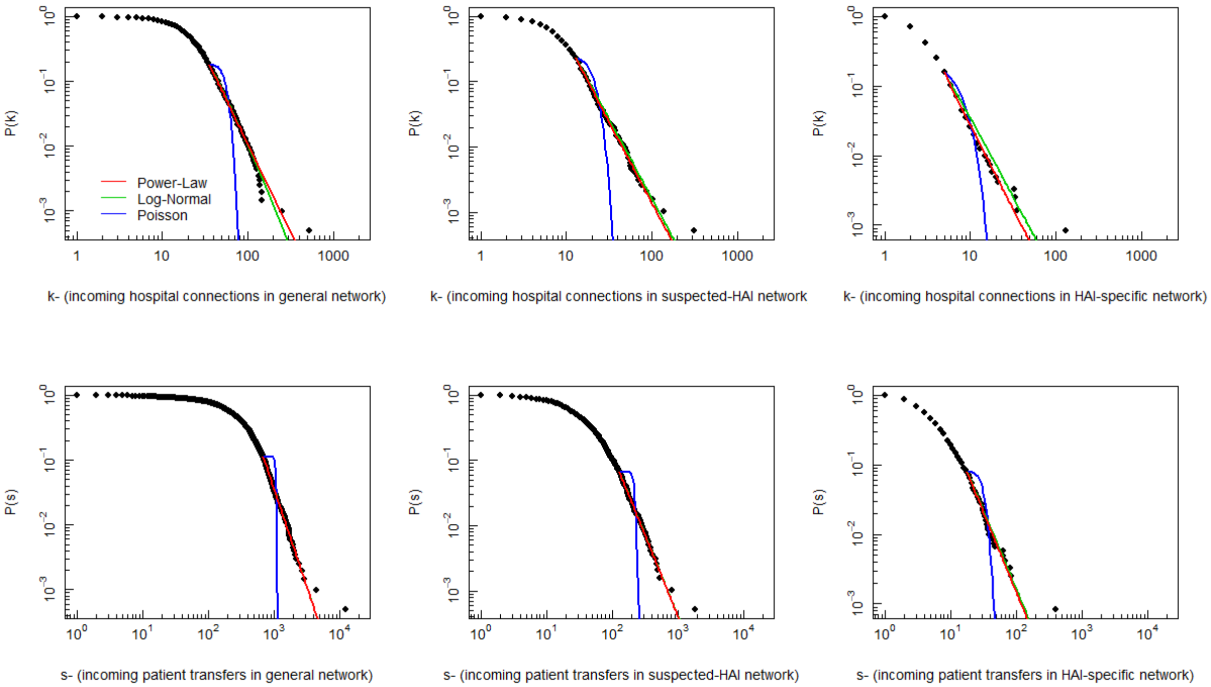

S5 Fig. The cumulative distribution functions of *k*- indegree for the general network (top left) and *s*- instrength (bottom left), suspected-HAI networks (top center, bottom center), and HAI-specific network (top right, bottom right). Fitted power-law (red), log-normal (green), and Poisson (blue) distributions are shown when: x-min for indegree = 36 and instrength = 698 in the general network; x-min for indegree = 13 and instrength = 131 in the suspected-HAI network; and x-min for indegree = 5 and instrength = 18 in the HAI-specific network. Power-law and log-normal had good fit for indegree and instrength in the three networks (KS-statistic p-values > 0.15) with the exception of log-normal distribution of indegree in the general and suspected-HAI network (KS-statistic p-value < 0.04). Poisson distribution was not a good fit for indegree and instrength in all networks (KS-statistic p-value < 0.0001).
