# Supplementary material for: Spread of hospital-acquired infections: A comparison of healthcare networks
Source: PLoS Comput Biol. 2017 Aug 24;13(8):e1005666. doi: 10.1371/journal.pcbi.1005666 (PMC5570216; doi:10.1371/journal.pcbi.1005666)
Supplement: S6 Fig — The cumulative distribution functions of k+ outdegree for the general network (top left) and s+ outstrength (bottom left), suspected-HAI networks (top center, bottom center), and HAI-specific network (top right, bottom right). Fitted power-law (red), log-normal (green), and Poisson (blue) distributions are shown when: x-min for outdegree = 101 and outstrength = 1102 in the general network; x-min for outdegree = 27 and outstrength = 70 in the suspected-HAI network; and x-min for outdegree = 7 and outstrength = 3 in the HAI-specific network. Only power-law distribution had a good fit for both outdegree and outstrength (KS-statistic p-values > 0.41) while log-normal distribution was only a good fit for the HAI-specific network (KS-statistic p-value = 0.15). (PDF) [file pcbi.1005666.s014.pdf]

S6 Fig. Cumulative Distribution Functions and Fit for Outdegree and Outstrength Distribution of the General, Suspected-HAI, and HAI-Specific Network

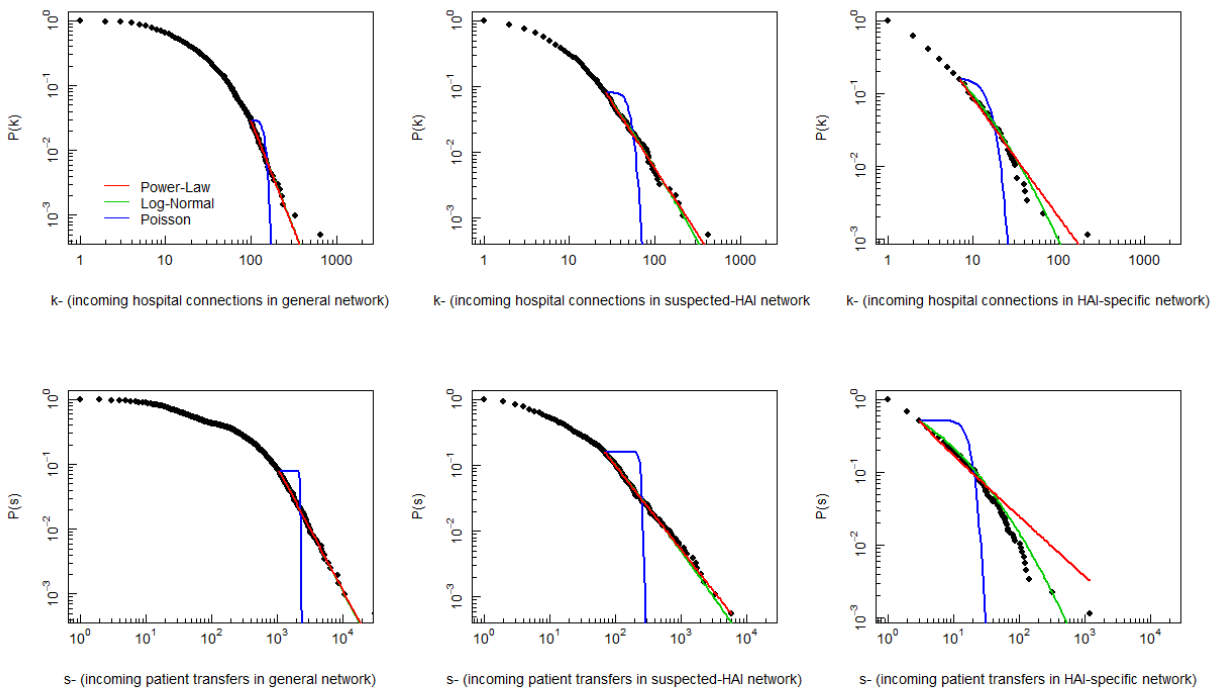

S6 Fig. The cumulative distribution functions of $k+$ outdegree for the general network (top left) and $s+$ outstrength (bottom left), suspected-HAI networks (top center, bottom center), and HAI-specific network (top right, bottom right). Fitted power-law (red), log-normal (green), and Poisson (blue) distributions are shown when: x-min for outdegree = 101 and outstrength = 1102 in the general network; x-min for outdegree = 27 and outstrength = 70 in the suspected-HAI network; and x-min for outdegree = 7 and outstrength = 3 in the HAI-specific network. Only power-law distribution had a good fit for both outdegree and outstrength (KS-statistic p-values > 0.41) while log-normal distribution was only a good fit for the HAI-specific network (KS-statistic p-value = 0.15).
