# Supplementary material for: Spread of hospital-acquired infections: A comparison of healthcare networks
Source: PLoS Comput Biol. 2017 Aug 24;13(8):e1005666. doi: 10.1371/journal.pcbi.1005666 (PMC5570216; doi:10.1371/journal.pcbi.1005666)
Supplement: S7 Fig — The length of the shortest paths or steps between any two nodes in the networks are calculated and plotted by their frequency. (PDF) [file pcbi.1005666.s015.pdf]

S7 Fig. Shortest path length distributions in the networks

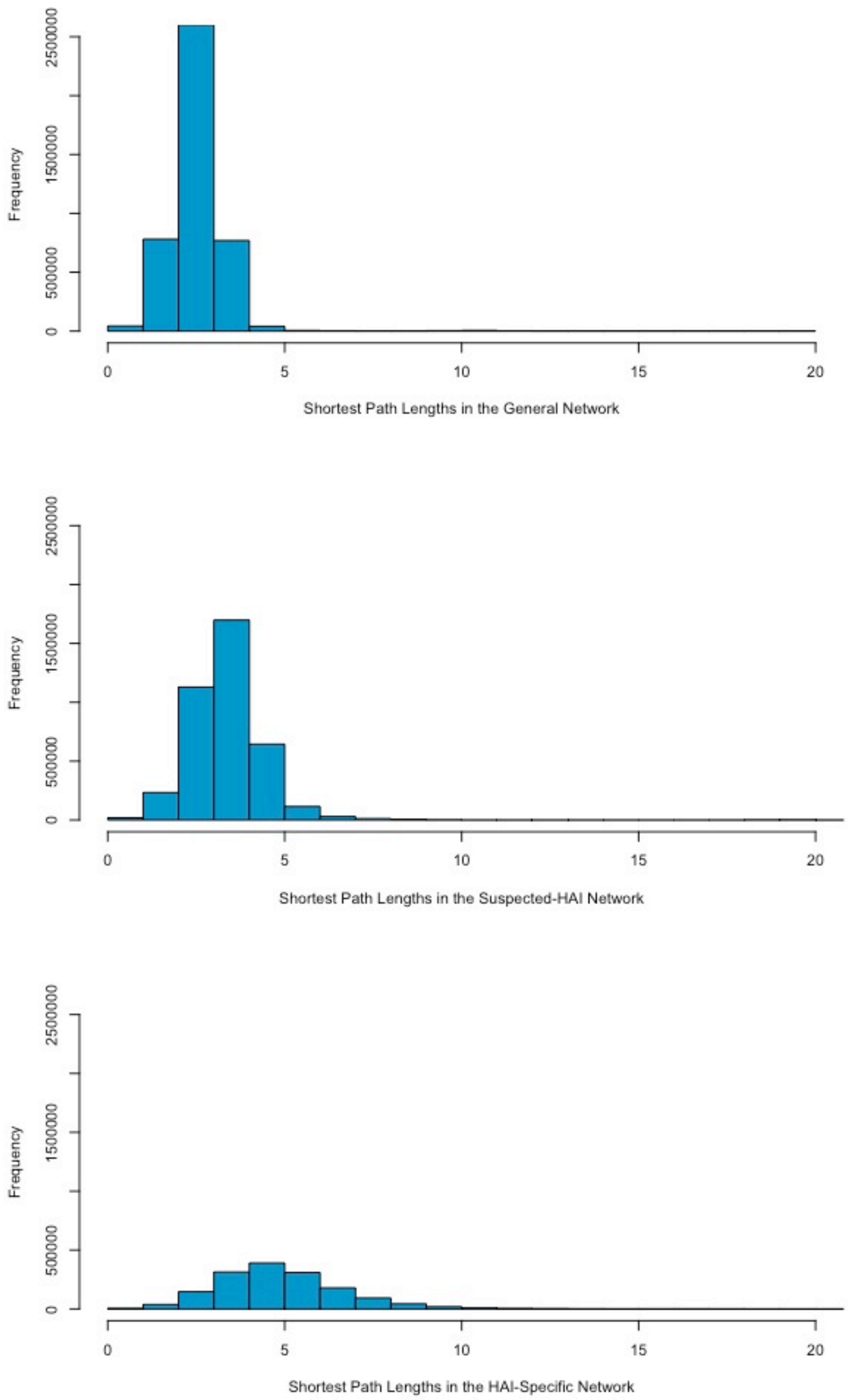

S7 Fig. The length of the shortest paths or steps between any two nodes in the networks are calculated and plotted by their frequency.
