# Supplementary material for: Spread of hospital-acquired infections: A comparison of healthcare networks
Source: PLoS Comput Biol. 2017 Aug 24;13(8):e1005666. doi: 10.1371/journal.pcbi.1005666 (PMC5570216; doi:10.1371/journal.pcbi.1005666)

S8 Fig. Distributions of p-values of hospital rank subsets using the Wilcoxon rank sum test in the HAI-specific network compared to the general network hospital ranks

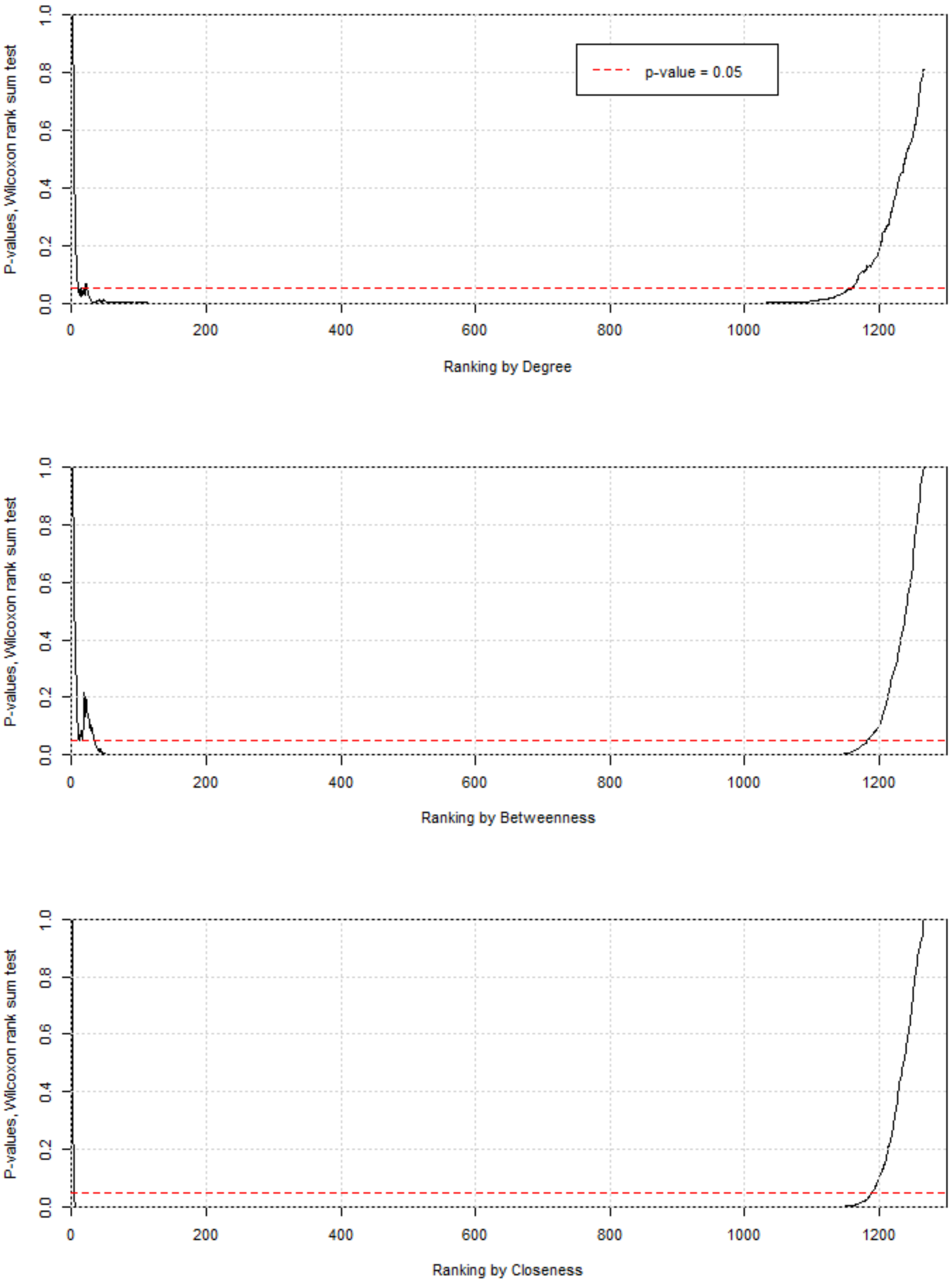

Supplement: S8 Fig — (PDF) [file pcbi.1005666.s016.pdf]
