# Supplementary material for: Spread of hospital-acquired infections: A comparison of healthcare networks
Source: PLoS Comput Biol. 2017 Aug 24;13(8):e1005666. doi: 10.1371/journal.pcbi.1005666 (PMC5570216; doi:10.1371/journal.pcbi.1005666)
Supplement: S10 Fig — Healthcare networks for all transferred patients (a) aged 18 and younger (b) aged 18 to 60 (c) older than 60 years old. Greedy communities were colored by the corresponding general network community regions with additional community color if not present for (a) 23 communities in which 3 were black (<5 hospitals) (b) 17 communities in which 1 was black (<5 hospitals) (c) 29 communities in which 14 were black (<5 hospitals). (PDF) [file pcbi.1005666.s018.pdf]

S10 Fig. Healthcare Networks by Age for All Patients

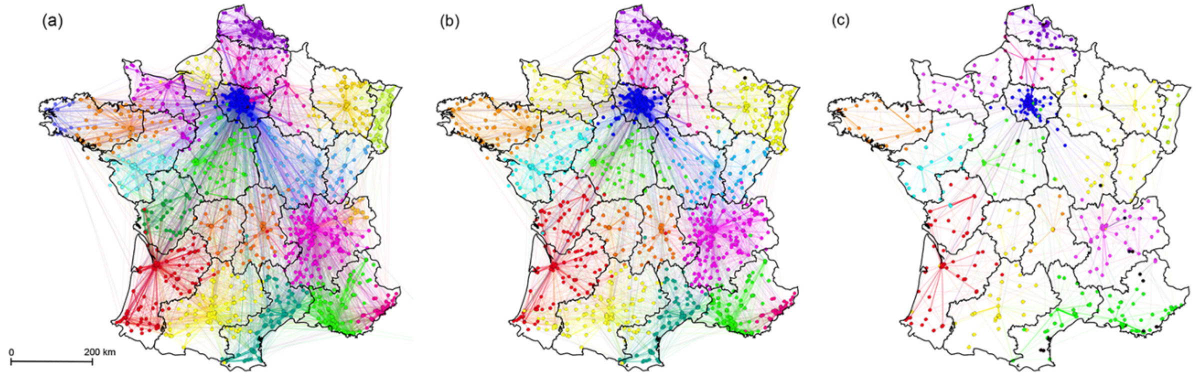

S10 Fig. Healthcare networks for all transferred patients (a) aged 18 and younger (b) aged 18 to 60 (c) older than 60 years old. Greedy communities were colored by the corresponding general network community regions with additional community color if not present for (a) 23 communities in which 3 were black (<5 hospitals) (b) 17 communities in which 1 was black (<5 hospitals) (c) 29 communities in which 14 were black (<5 hospitals).
