# Supplementary material for: Spread of hospital-acquired infections: A comparison of healthcare networks
Source: PLoS Comput Biol. 2017 Aug 24;13(8):e1005666. doi: 10.1371/journal.pcbi.1005666 (PMC5570216; doi:10.1371/journal.pcbi.1005666)
Supplement: S11 Fig — Healthcare networks for all transferred patients (a) aged 18 and younger (b) aged 18 to 60 (c) older than 60 years old. We detected a total number of Greedy-based communities for each age network (a) 22 total and 19 with over 2 hospitals from a network of 1894 hospitals and 11234 edges (b) 30 total with 17 with over 2 hospitals from a network of 1559 hospitals and 5423 edges (c) 29 total with 14 with over 2 hospitals per community from a network of 218 hospitals and 318 trajectories. (PDF) [file pcbi.1005666.s019.pdf]

S11 Fig. Healthcare Networks by Age for Suspected-HAI Patients

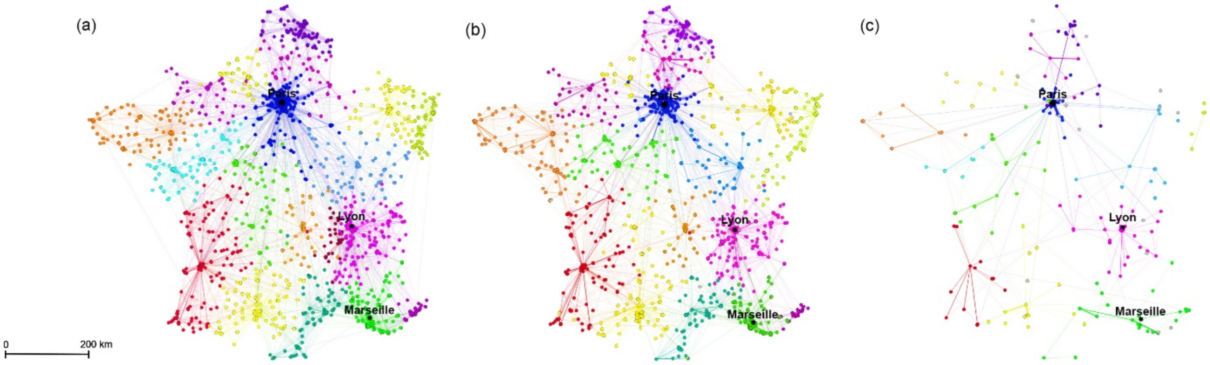

S11 Fig. Healthcare networks for all transferred patients (a) aged 18 and younger (b) aged 18 to 60 (c) older than 60 years old. We detected a total number of Greedy-based communities for each age network (a) 22 total and 19 with over 2 hospitals from a network of 1894 hospitals and 11234 edges (b) 30 total with 17 with over 2 hospitals from a network of 1559 hospitals and 5423 edges (c) 29 total with 14 with over 2 hospitals per community from a network of 218 hospitals and 318 trajectories.
