# Supplementary material for: Spread of hospital-acquired infections: A comparison of healthcare networks
Source: PLoS Comput Biol. 2017 Aug 24;13(8):e1005666. doi: 10.1371/journal.pcbi.1005666 (PMC5570216; doi:10.1371/journal.pcbi.1005666)
Supplement: S12 Fig — Healthcare networks for all transferred patients (a) aged 18 and younger (b) aged 18 to 60 (c) older than 60 years old. Network communities are detected using the Greedy algorithm and colored according to community membership for (a) 1143 hospitals and 2260 edges with 50 total communities with only 28 composed of more than 5 hospitals (b) 603 hospitals and 593 edges with 41 total communities and 19 with over 5 hospitals (c) 44 hospitals and 33 edges, 18 total communities, 2 communities with more than 5 hospitals, and 9 communities with more than 2 hospitals. (PDF) [file pcbi.1005666.s020.pdf]

S12 Fig. Healthcare Networks by Age for HAI-Specific Patients

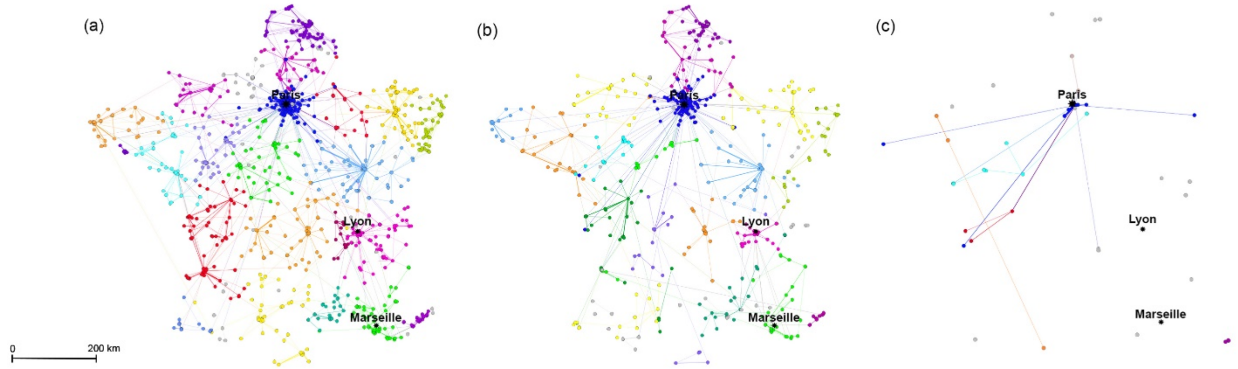

S12 Fig. Healthcare networks for all transferred patients (a) aged 18 and younger (b) aged 18 to 60 (c) older than 60 years old. Network communities are detected using the Greedy algorithm and colored according to community membership for (a) 1143 hospitals and 2260 edges with 50 total communities with only 28 composed of more than 5 hospitals (b) 603 hospitals and 593 edges with 41 total communities and 19 with over 5 hospitals (c) 44 hospitals and 33 edges, 18 total communities, 2 communities with more than 5 hospitals, and 9 communities with more than 2 hospitals.
