# Supplementary material for: Spread of hospital-acquired infections: A comparison of healthcare networks
Source: PLoS Comput Biol. 2017 Aug 24;13(8):e1005666. doi: 10.1371/journal.pcbi.1005666 (PMC5570216; doi:10.1371/journal.pcbi.1005666)
Supplement: S1 Table — Comparison of the healthcare network topology measures with the average measures of 100 simulated Erdos-Renyi (ER) networks that are parameterized with same number of nodes, edges, and Poisson-distributed average edge weight. For each measure, a t-test is conducted to compare the difference between the health network value and the average values of the ER networks with given 95% confidence intervals and p-values. (PDF) [file pcbi.1005666.s021.pdf]

***S1 Table. Network Characteristics of the Erdos-Renyi Random Networks***

| | HAI-Specific Network | 100 HAI-Specific-like ER networks | | | Suspected-HAI Network | 100 Suspected-HAI-like ER networks | | | General Network | 100 General Network-like networks | | |
|---|---|---|---|---|---|---|---|---|---|---|---|---|
| **Network Topology Measures** | Value | Mean | 95% CI | P-value | Value | Mean | 95% CI | P-value | Value | Mean | 95% CI | P-value |
| Average Degree | 5.88 | 5.88 | 5.88-5.88 | 1 | 19.05 | 19.05 | 19.05-19.05 | 1 | 48.50 | 48.50 | 48.50-48.50 | 1 |
| Diameter | 47 | 37.31 | 36.64-37.98 | <0.001 | 64 | 27.83 | 27.43-28.23 | <0.001 | 30 | 49.87 | 49.69-50.05 | <0.001 |
| Average Path Length | 5.23 | 6.55 | 6.54-6.56 | <0.001 | 3.63 | 3.618 | 3.618-3.619 | <0.001 | 2.99 | 2.735 | 2.735-2.735 | <0.001 |
| Global Clustering Coefficient | 0.08 | 0.0047 | 0.0045-0.0048 | <0.001 | 0.16 | 0.0096 | 0.0095-0.0097 | <0.001 | 0.23 | 0.02337 | 0.02334-0.0234 | <0.001 |
| Density | 0.002 | 0.002 | 0.002-0.002 | 1 | 0.005 | 0.0048 | 1975-1975 | 1 | 0.012 | 0.012 | 0.012-0.012 | 1 |
| Average Edge Betweenness | 1556.94 | 3143.72 | 3128.89-3158.55 | <0.001 | 852.23 | 989.39 | 988.07-990.70 | <0.001 | 301.27 | 252.81 | 252.75-252.88 | <0.001 |
| Average Total Closeness | 3.2e-5 | 9.3e-5 | 8.9e-5-9.7e-5 | <0.001 | 7.4e-5 | 5.8e-5 | 5.79e-5-5.81e-5 | <0.001 | 1.6e-4 | 1.89e-5 | 1.89e-5-1.90e-5 | <0.001 |

**S1 Table.** Comparison of the healthcare network topology measures with the average measures of 100 simulated Erdos-Renyi (ER) networks that are parameterized with same number of nodes, edges, and Poisson-distributed average edge weight. For each measure, a t-test is conducted to compare the difference between the health network value and the average values of the ER networks with given 95% confidence intervals and p-values.
